# Green synthesis of novel stable biogenic gold nanoparticles for breast cancer therapeutics via the induction of extrinsic and intrinsic pathways

**DOI:** 10.1038/s41598-022-15648-y

**Published:** 2022-07-07

**Authors:** Nehal M. El-Deeb, Sara M. Khattab, Morsy A. Abu-Youssef, Ahmed M. A. Badr

**Affiliations:** 1 Biopharmaceutical Products Research Department, Genetic Engineering and Biotechnology Research Institute, City of Scientific Research and Technological Applications (SRTA-City), P.O. Box 21934, New Borg El-Arab City, Alexandria Egypt; 2 Pharmaceutical and Fermentation Industries Development Center, City of Scientific Research and Technological Applications (SRTA-City), P.O. Box 21934, New Borg El-Arab City, Alexandria Egypt; 3 Chemistry Department, Faculty of Science, Alexandria University, P.O. Box 426, Ibrahimia, Alexandria 21321 Egypt

**Keywords:** Breast cancer, Cancer, Chemistry, Biosynthesis

## Abstract

Biosynthesis of gold nanoparticles (AuNPs) using algal polysaccharides is a simple, low-cost, and an eco-friendly approach. In the current study, different concentrations of *Arthospira platensis* exopolysaccharides (EPS) were used to synthetize AuNPs via the reduction of gold ions. The biologically synthesized AuNPs (AuNPs1, AuNPs2, AuNPs3) were prepared in 3 different forms through the utilization of three different ratios of EPS-reducing agents. AuNPs analysis confirmed the spherical shape of the EPS-coated AuNPs. Furthermore, AuNPs prepared by EPS and l-ascorbic acid (AuNPs3) showed more stability than the AuNPs colloidal solution that was prepared using only l-ascorbic acid. Analysis of the antimicrobial effects of AuNPs showed that *E. coli* was the most sensitive bacterial species for AuNPs3 and AuNPs1 with inhibition percentages of 88.92 and 83.13%, respectively. Also, safety assay results revealed that AuNPs3 was the safest biogenic AuNPs for the tested noncancerous cell line. The anticancer assays of the biogenic AuNPs1, AuNPs2, and AuNPs3 against MCF-7 cell line indicated that this cell line was the most sensitive cell line to all treatments and it showed inhibition percentages of 66.2%, 57.3%, and 70.2% to the three tested AuNPs, respectively. The AuNPs also showed abilities to arrest MCF-7 cells in the S phase (77.34%) and increased the cellular population in the sub G0 phase. Gene expression analysis showed that AuNPs3 down regulated Bcl2, Ikapα, and Survivn genes in MCF-7 treated-cells. Also, transmission electron microscopy (TEM) analysis of MCf-7 cells revealed that AuNPs 3 and AuNPs2 were localized in cell vacuoles, cytoplasm, and perinuclear region.

## Introduction

Recently, Nanotechnology term has become more interesting than before and gained credit from its raising importance during the past few decades. Nanotechnology provides advanced strategies against cancer and could minimize the chemotherapeutic drug-induced adverse effects^1^. FDA hasapproved some anticancer drugs, diagnostic, and/or targeting agents such as nanobiomaterials conjugates to combat cancer cells^2^. In addition to the usual liposomes and dendrimers nano-platforms that are used in cancer therapeutics, silver and gold nano biomaterials have gained more significant attention in cancer diagnosis and therapies due to their unique physicochemical properties^2^. Gold nanoparticles (AuNPs) play a significant role in the nanotechnology field due to their potential applications in many important fields including; optics, catalysis, and many medical applications. Their good biocompatibility, possible solubility in aqueous phases, and their photonic properties enabled the integration of AuNPs in diverse biomedical fields^3–7^. As anticancer agents, AuNPs considered promising agents in competing different types of cancers such as prostate cancer^8^, breast cancer^2^, and colorectal cancer^9^. Also, the antibacterial activities of AuNPs were confirmed against different pathogenic bacteria such as; *Staphylococcus epidermidis*, *Escherichia coli*^10^, *Corynebacterium pseudotuberculosis*^11^, Gram-positive bacterial strain methicillin-sensitive *Staphylococcus aureus* ATCC 29213 (MSSA), methicillin-resistant *Staphylococcus aureus* ATCC 43300 (MRSA), Gram-negative *E. coli* ATCC 25922 (EC), and a clinical isolate of *E. coli* 11046 (CI-EC)^12^. Although the significant biomedical and pharmaceutical potentials of the biogenic metals nanoparticles, some hazards have been reviewed^13^ to be associated with the use of the non-biogenic metals nanoparticles, which were prepared by different physical and/or chemical processes. So that, there is an increasing demand for adapting nontoxic, high yield, cost effective, eco-friendly, and environmentally safe methods for synthesizing different types of metal nanoparticles^14^. Many biological sources that normally exist in our environment could be used in the biosynthesis of biogenic nanoparticles such as; algae, cyanobacteria, fungi, actinomycetes, yeast, bacteria, plants, and viruses. In addition to a plethora of their metabolites such as; proteins, polysaccharides, lipids, terpenoids, flavonoids, amines, amides, carbonyl groups, phenolics, proteins, pigments, alkoids, and plenty of reducing agents that exist in plant and microbial extracts^15–19^. Different biological agents have been used in the production of AuNPs intracellularly including yeast^20^, fungi^21^, and bacteria^22^. Biosynthesis of gold nanoparticles could also occur extracellularly, such as in the case of *Aloe vera*^19^, *Cinnamomum camphora*^23^, *Medicago sativa*^24^, *Azadirachta indica*^25^, *Tamarindus indica*^14^ and *Pelargonium graveolens*^26^.

Spirulina *(Arthospira platensis)* is a filamentous *cynanobacterium* (Oscillatoriaceae) that shows great plasticity as it has a soft cell wall made of complex sugars and proteins^27^. Several factors contribute to their morphological features such as: temperature, physical and chemical factors, and genetic material alteration. Spirulina forms helical trichomes of variable sizes and degrees of coiling. These trichomes can be tightly coiled or even straight uncoiled^28,29^. This microalga has been reported to be used as food since 1521^28^. At present days, Spirulina is considered as one of the most important sources of vitamins, essential fatty acids, and other biologically useful substances. Also, it contains up to 70% of vegetable proteins, a good balance in amino acids, and highly enriched with beta carotene and iron^30,31^*.* Polysaccharides mainly have large and complex molecular structures, which consist of different monosaccharides linked together via glycosidic bonds^32^. Spirulina extracted-polysaccharides can act as antioxidant agents. These polysaccharides prevent oxidation of cellular substrates and maintain cellular homeostasis by removing free radicals^33,34^, which can lead to different degenerative diseases such as cancer, coronary heart disease, Alzheimer's, neurodegenerative disorders, atherosclerosis, diabetes, aging, cataracts and various inflammations^35,36^. Also, Spirulina extracted-polysaccharides can intensify non-specific cellular immune functions inside the body that help in viruses resistance^37^.

In the current study, we are examining, for the first time, the use of *Arthospira platensis* exo*-*polysaccharides in the biosynthesis of gold nanoparticles using a low-cost and an eco-friendly system. In addition, we are investigating the structural properties and the biological activities of the newly biosynthesized nanoparticles against different microbial strains and cancer cell lines as antimicrobial and anticancer agents, respectively.

## Results

### Green synthesis of AuNPs

Biologically synthesized AuNPs were obtained in 3 different forms using different ratios of the tested reducing agents. The recovered NPs were; AuNPs1 (1:1 molar ratio of NaAuCl_4_: polysaccharides); AuNPs2 (2:1 molar ratio of NaAuCl_4_: polysaccharides); and AuNPs3 (1:1 molar ratio NaAuCl_4_: polysaccharides and reduced by l-ascorbic acid).

### Characterization of AuNPs

#### AuNPs stability using UV–Vis spectral analysis

**Figure 1:**
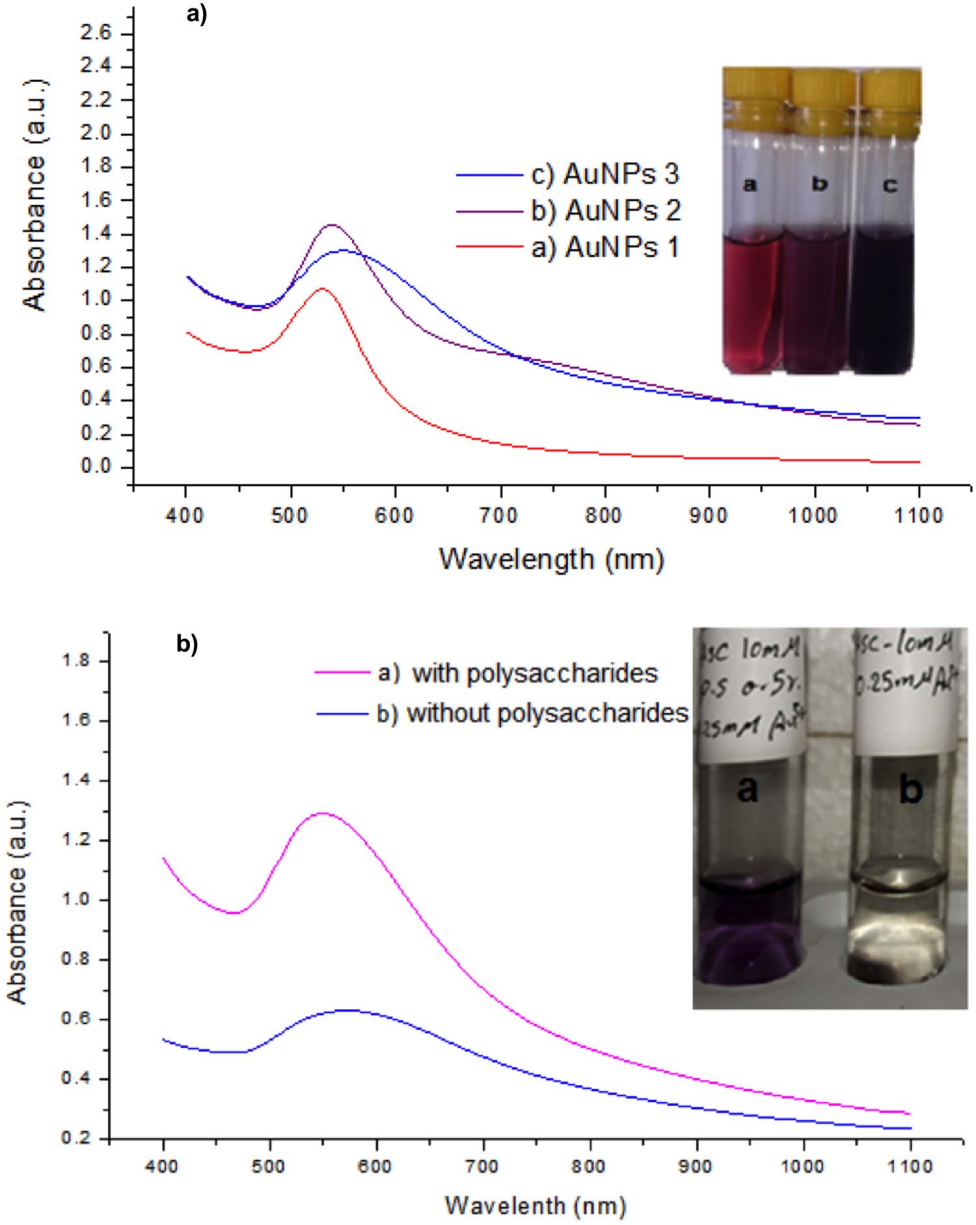
UV–Vis spectral analysis of AuNpPs. (**a**) UV–Vis spectroscopic measurements of the 4 forms of the biogenic nanoparticles; AuNPs1, AuNPs2 and AuNPs3. (**b**) The UV–Visible spectrum of AuNPs after 3 months that prepared by polysaccharides with l-ascorbic acid (AuNPs 3), AuNPs3 were more stable even after more than 3 months comparing with nanogold colloidal solution that prepared by l-ascorbic acid alone as a reducing and stabilizing agent.

The obtained data illustrated in Fig. 1a display the absorption peaks of the three different samples of gold nanoparticles (AuNPs1, AuNPs2, AuNPs3). The three spectra were normalized at the maximum Plasmon absorptions at 530.0, 540.0, and 550.0 nm. The aggregation state was detected visually by observing the change in solution color from red to blue or purple. Blue shift was observed here from 550 to 530 nm. Furthermore, the UV–Vis spectrum of AuNP3 that were prepared with polysaccharides and l-ascorbic acid was staple and didn’t record any significant changes even after more than 3 months comparing with nanogold colloidal solution, which was prepared with only l-ascorbic acid as a reducing and stabilizing agent (Fig. 1b).

#### X-ray powdered diffraction (XRD)

**Figure 2:**
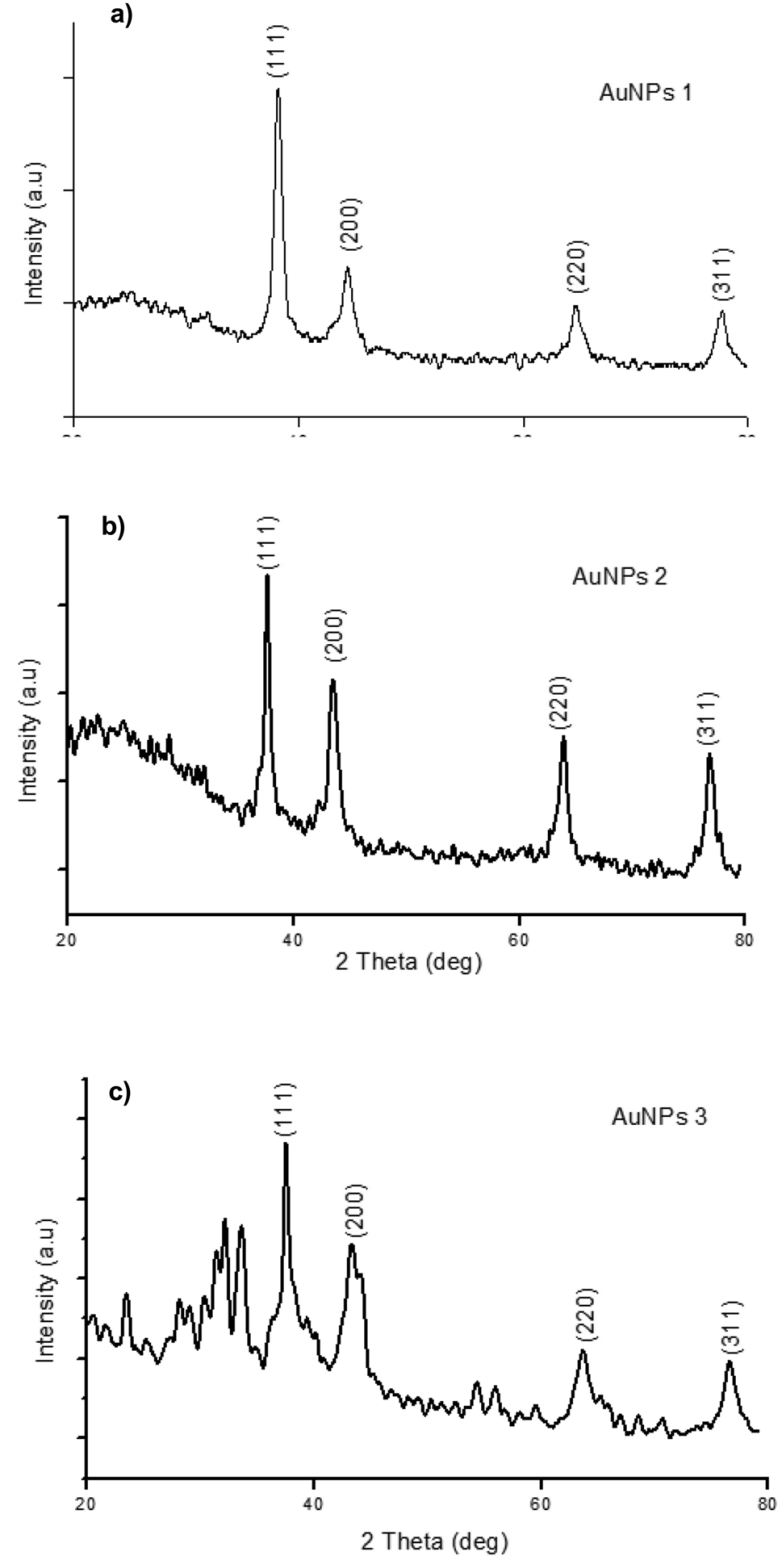
X-ray powdered diffraction of AuNPs. X-ray powdered diffraction of AuNPs1 (**a**), AuNPs2 (**b**) and AuNPs3 (**c**).

AuNPs were investigated using XRD to confirm their crystalline structures, where all of them expressed the most characteristic peaks of metallic gold. Four intensive Bragg reflections were observed in each case around 38.0996°, 44.3687°, 64.6765°, and 77.5471° corresponding to Miller indices (1 1 1), (2 0 0), (2 2 0), and (3 1 1). This confirmed the face-centered cubic crystalline symmetry of gold nanoparticles (JCPDS file no. 01-1174). The ratios between the intensities of (2 0 0) and (1 1 1) diffraction peaks were 0.31 in case of AuNPs1, 0.26 in case of AuNPs2, and 0.32 in case of AuNPs3 (Fig. 2a,b,c). All intensity ratios were lower than the conventional bulk intensity ratio of ~ 0.52. These findings confirmed that (1 1 1) is the preferential or predominant orientation, as confirmed previously by the following TEM studies. Nanoparticles sizes were estimated using XRD measurements (Table 1) by applying Debye Scherrer equation.

**Table 1:** The recorded results from applying Scherrer equation for prepared NPs sizes:

Nanoparticles sample	Average size of particles (nm)
(a) AuNPs1	12.13 ± 0.003
(b) AuNPs2	15.10 ± 0.013
(c) AuNPs3	10.50 ± 0.024

#### Transmission electron microscopy (TEM)

**Figure 3:**
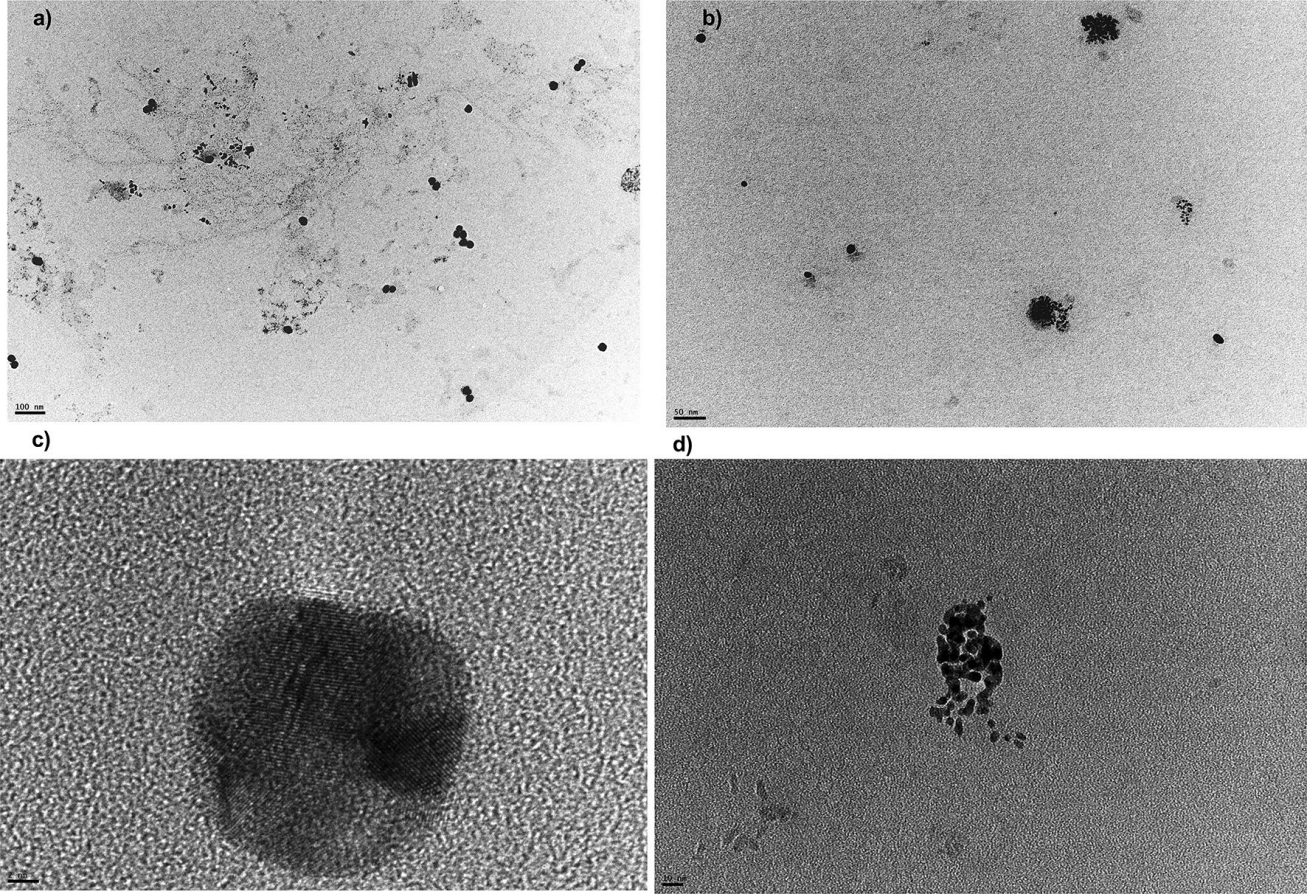
Transmission electron microscopy (TEM) of AuNPs. TEM images for biogenic prepared nanoparticles (**a**) AuNPs1, (**b**) AuNPs2, (**d**) AuNPs 3. The high-resolution TEM image is showing a single nanocrystal for AuNPs1 (**c**).

The morphology of the biogenic AuNPs was investigated using TEM scaning. The results revealed that spherical shape was the predominant shape in all the AuNPs preparations . AuNPs1 particle size that was estimated from TEM analysis ranged from 9.0 to 30.0 nm (Fig. 3). While, AuNPs2 showed a range of particle sizes between 8.0 and 35.0 nm, and AuNPs3 have particle sizes ranged from 6.0 to 40.0 nm (Fig. 3b).

High-resolution TEM imaging indicated that AuNPs1 showed only a single nano-crystal structure that appeared with clear lattice fringes with spacing of 0.234 nm (Fig. 3c). This result confirmed that nanogold crystals grow preferentially on the (1 1 1) plane (Fig. 2). This was confirmed by the d-spacing of (1 1 1) plane (provided from XRD measurement), which is equal to 0.2364 nm and the calculated interplanar distance of Au (1 1 1) plane.

#### FTIR analysis

##### FTIR analysis of algal polysaccharides

FTIR analysis was used to analyze the functional groups of the extracted polysaccharides and to identify which biomolecules are responsible for the reduction and capping of the prepared biogenic gold nanoparticles.

**Figure 4:**
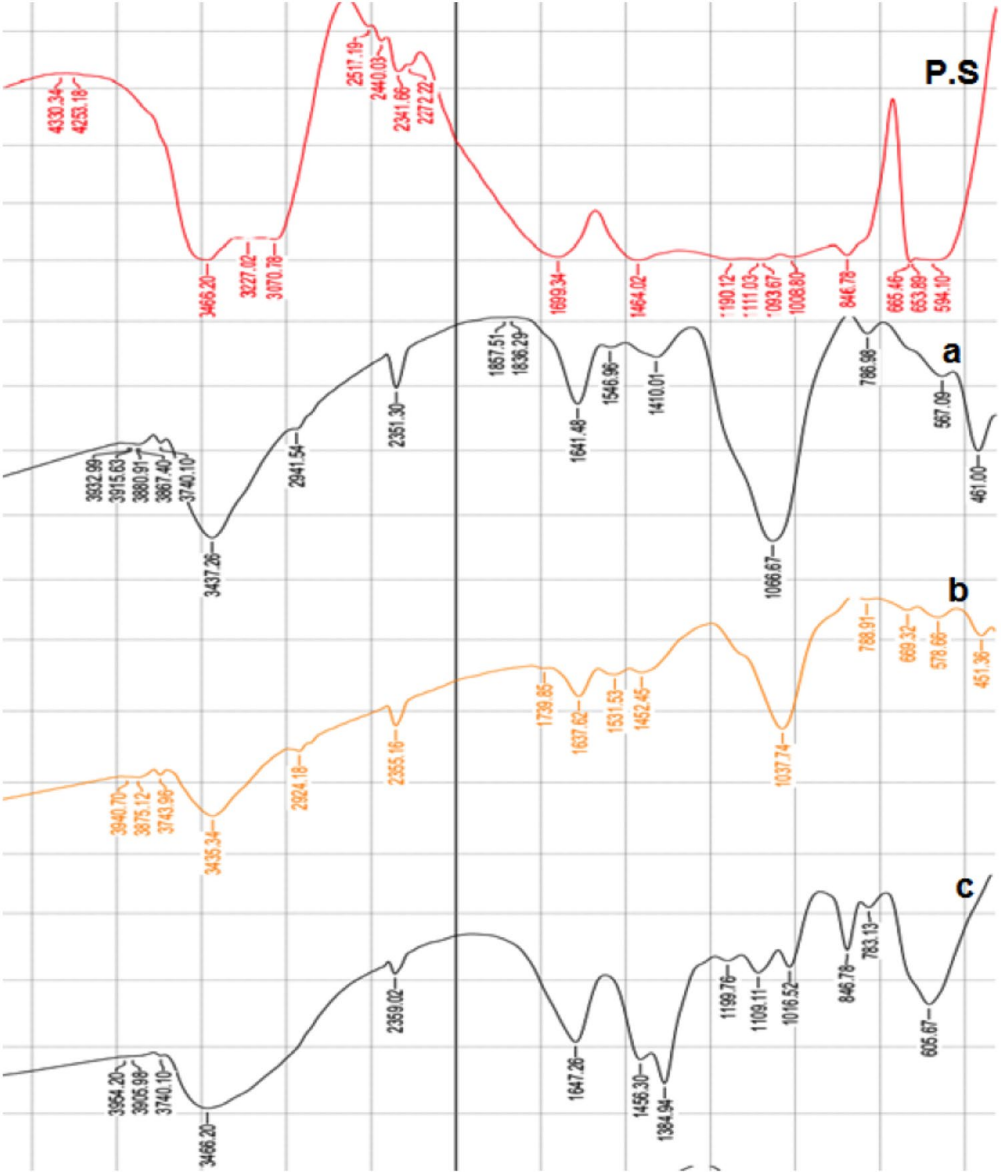
FTIR analysis of Algal polysaccharides and AuNPs. FTIR measurements were used to analyze the functional groups present in the extracted polysaccharides (P.S) and to identify which biomolecules are responsible for the reduction and capping of AuNPs1 (**a**), AuNPs2 (**b**) and AuNPs3 (**c**).

The FTRI spectrum of the extracted polysaccharides showed peaks at 1464.0, 1699.0, 3070.0, and 3466.0 cm^−1^. In addition, multiple peaks were observed at 665.0, 846.0, and 1008.0–1190.0 cm^−1^. A broad intense band at 3466.0 cm^−1^ was also observed, which could be assigned to OH groups of algal polysaccharides or a secondary amide group (Fig. 4, upper panel). The band detected at 3070.0 cm^−1^ could be assigned to alkenyl =C—H stretching or to N—H of a secondary amine. The absorption band observed at 1699.0 cm^−1^ could be assigned to the carbonyl groups, stretching groups of algal polysaccharides, or to the amide groups of algal proteins. The band at 1464.0 cm^−1^ could be assigned to the –COO^−^ groups. The detected multiple peaks between 1008.0 and 1190.0 cm^−1^, which are characteristic for sugars moieties that could be due to the coupling of the C–O or C–C stretching modes with the C–O–H bending modes. The stretching vibrations of C—O can be triggered from different sources such as carboxylic acid or polyol, where the extracted polysaccharides have a variety of components like neutral sugars, uronic acids, and amino sugars. The band observed at 846.0 cm^−1^ can be assigned to–C—O—SO_4_ of sulfated polysaccharides. Finally, the band at 665.0 cm^−1^ can be assigned to SO_4_^−2^ groups.

##### FTIR analysis of biogenic AuNPs nanoparticles

FTIR analysis of the prepared biogenic AuNPs revealed the presence of absorption peaks at 1035.0, 1450.0, 1540.0, 1640.0, and 3445.0 cm^−1^. The broad peak at nearly 3445.0 cm^−1^ in the nanoparticles spectra can be assigned to OH group (Fig. 4a). This indicates that the presence of OH groups in the polysaccharides is the key group involved in the reduction of Au ions. The absorption band at about 1640.0 cm^−1^ can be assigned to the stretching vibration of –C = O of associated secondary amide groups^38^. The two observed peaks at nearly 1540.0 and 1450.0 cm^−1^ can be assigned to the stretching vibration of –COO^−^ groups (Fig. 4b,c). The band at about 1035.0 cm^−1^ can be assigned to the stretching vibrations of C—N groups of aromatic amines or to the stretching of S = O of sulfated extracted polysaccharides. The only exception was for sample (C), where it showed a unique peak at 1384.0 cm^−1^ that can be assigned to C—H deformations of –CH_3_ or –CH_2_ groups.

### Biological activities of the biogenic AuNP

#### Antimicrobial activity of the biogenic AuNPs

**Figure 5:**
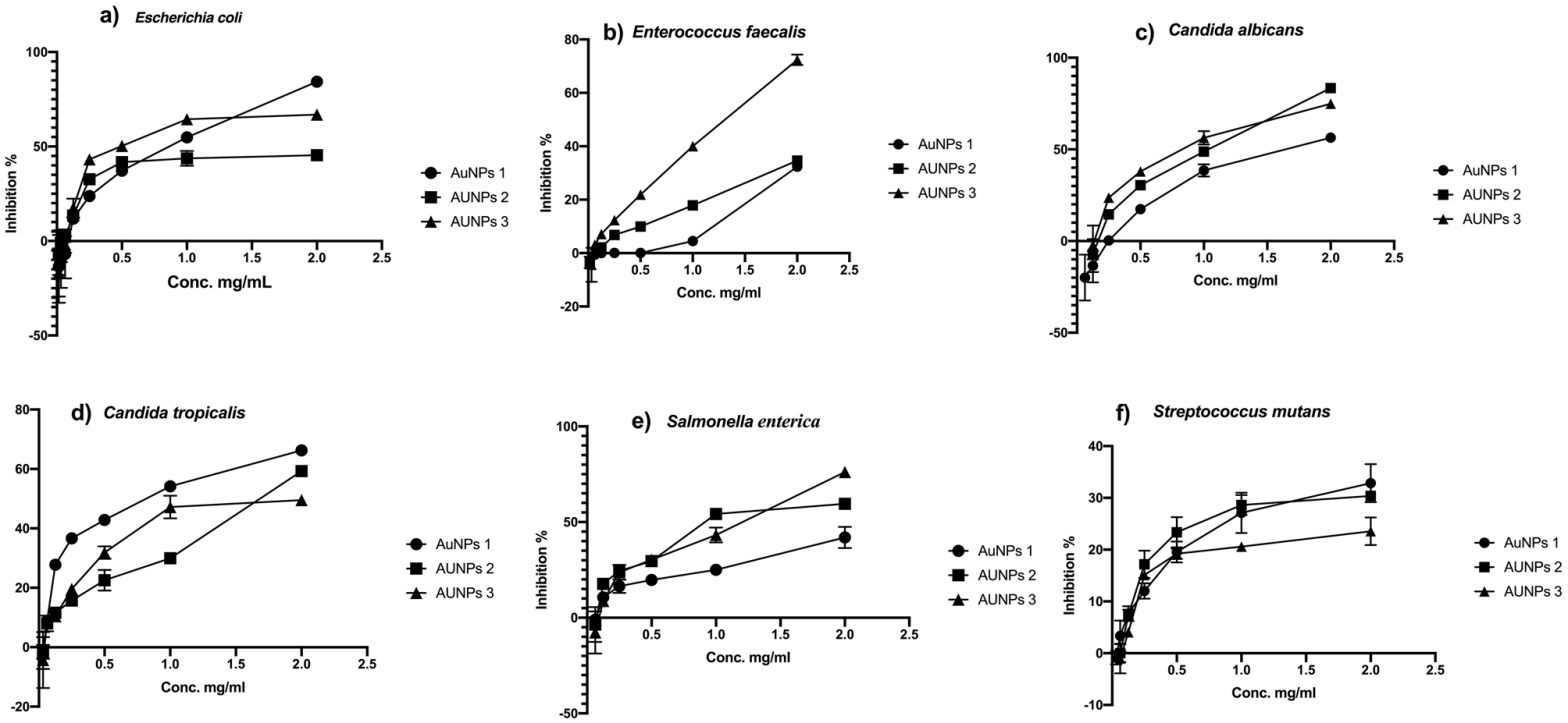
Antimicrobial Activities of the biogenic AuNPs. The antibacterial activity of the biogenic AuNPs1,2,3 were tested on *Escherichia coli* (**a**), *Enterococcus faecalis* (**b**)*, Candida albicans* (**c**), *Candida tropicalis* (**d**), *Salmonella enterica* (**e**), and *Streptococcus mutans* (**f**). The antimicrobial activity were quantified after 24 h. and expressed as inhibition percentage comparing with the non-treated strains.

**Figure 6:**
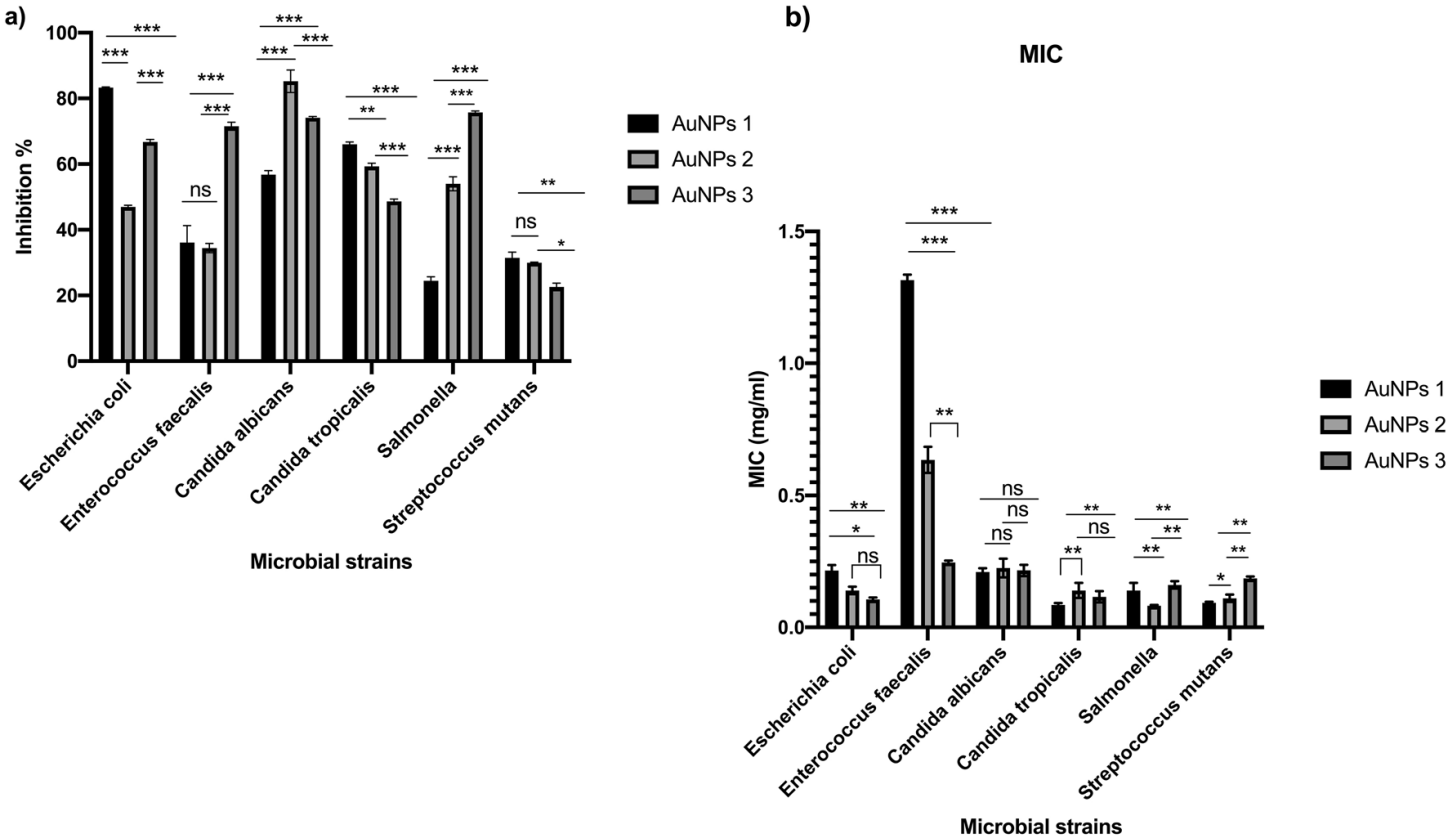
The maximum inhibition percentage values and MIC values of the biogenic AuNPs. The maximum antibacterial activity of the biogenic AuNPs1,2,3 were calculated against *Escherichia coli*, *Enterococcus faecalis, Candida albicans*, *Candida tropicalis*, *Salmonella enterica*, and *Streptococcus mutans* (**a**). The minimum inhibitory concentrations of AuNPs1,2,3 were calculated on the tested strains using the different concentrations of the treatments (**b**).

Antibacterial activity of the biogenic AuNPs was tested on *E. coli*, *Enterococcus faecalis, Candida albicans*, *Candida tropicalis*, *Salmonella enterica*, and *Streptococcus mutans*. The results of microplate assay indicated that *E. coli* was the most sensitive bacterial species for both AuNPs3 and AuNPs1 treatments with inhibition percentages of 88.92 and 83.13%, respectively (Figs. 5a, 6a). In addition, AuNPs3 recorded the lowest MIC value on *E. coli*100.0 µg/ml (Fig. 6b). Concerning *C. albicans*, its growth was greatly inhibited with AuNPs2 treatment, which showed inhibition percentage that reached 82.83% (Fig. 5c). In addition, both AuNPs1 and AuNPs2 showed moderate to high inhibition percentages for *C. tropicalis* growth with values of 65.51 and 58.57%, respectively (Fig. 5d). Furthermore, both *E. faecalis* and *S. enterica* were sensitive to AuNPs3 with inhibition percentages of 73.76 and 75.35%, respectively (Fig. 5b,e). *S. mutans* was the most resistant strain to AuNPs treatment with maximum inhibition percentage < 40.0% (Figs. 5f, 6a). The lowest MIC was recorded on *C. tropicalis* upon treatment with AuNPs with value 90.0 µg/ml (Table 2, Fig. 6b).

**Table 2:** The recorded MIC values of the biogenic AuNPs against the tested microbial strains.

Microbial strain	MIC (mg/mL)		
	AuNPs1	AuNPs2	AuNPs3
*Escherichia coli*	0.215 ± 0.02	0.14 ± 0.014	0.105 ± 0.007
*Enterococcus faecalis*	1.315 ± 0.021	0.635 ± 0.049	0.245 ± 0.007
*Candida albicans*	0.21 ± 0.014	0.225 ± 0.0353	0.215 ± 0.021
*Candida tropicalis*	0.085 ± 0.007	0.14 ± 0.0282	0.115 ± 0.0221
*Salmonella*	0.14 ± 0.028	0.082 ± 0.002	0.16 ± 0.014
*Streptococcus mutans*	0.093 ± 0.004	0.11 ± 0.014	0.185 ± 0.007

#### Safety pattern of the biogenic AuNPs

**Figure 7:**
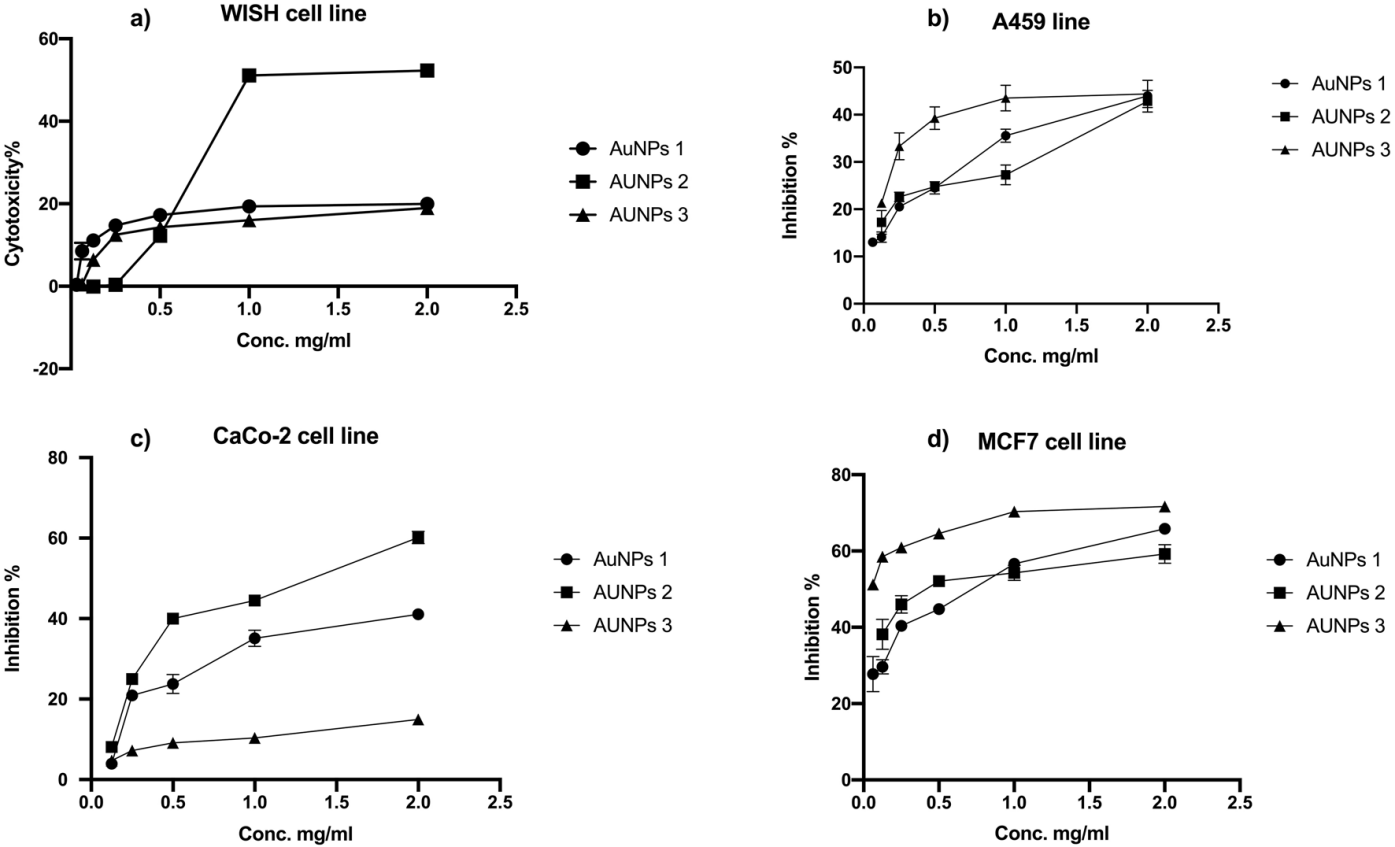
Safety assays and anticancer activity of AuNPs. The safety assay of AuNPs on the non-cancerous cell line (WISH cells) using different concentrations 2.00 to 0.25 mg/ml by MTS assay (**a**). the anticancer effects of AuNPs1,2,3 were tested against A549 (**b**), CaCo-2 (**c**) and MCF-7 cell lines (**d**) were investigated using MTS assay protocol using different concentrations 2.00 to 0.25 mg/ml.

In vitro viability test was used to investigate the safety patterns of the AuNPs at different concentrations (2.0 to 0.0156 mg/ml). WISH cell line was used as a non-cancerous cell line model to detect the AuNPs safest doses to be used in the proceeding tests. Using MTs assay, the safest AuNPs preparation was found to be AuNPs3 that showed 17.35% maximum toxicity. While, both AuNPs1 and AuNPs2 recorded 18.92% and 51.4% cytotoxic percentages on WISH cells, respectively. All AuNPs recorded more than 90.0% cellular viability (Fig. 7a) at a concentration of 0.031 mg/ml.

#### In vitro anticancer activities of the biogenic AuNPs

##### Cytotoxicity assay of the biogenic AuNPs

**Figure 8:**
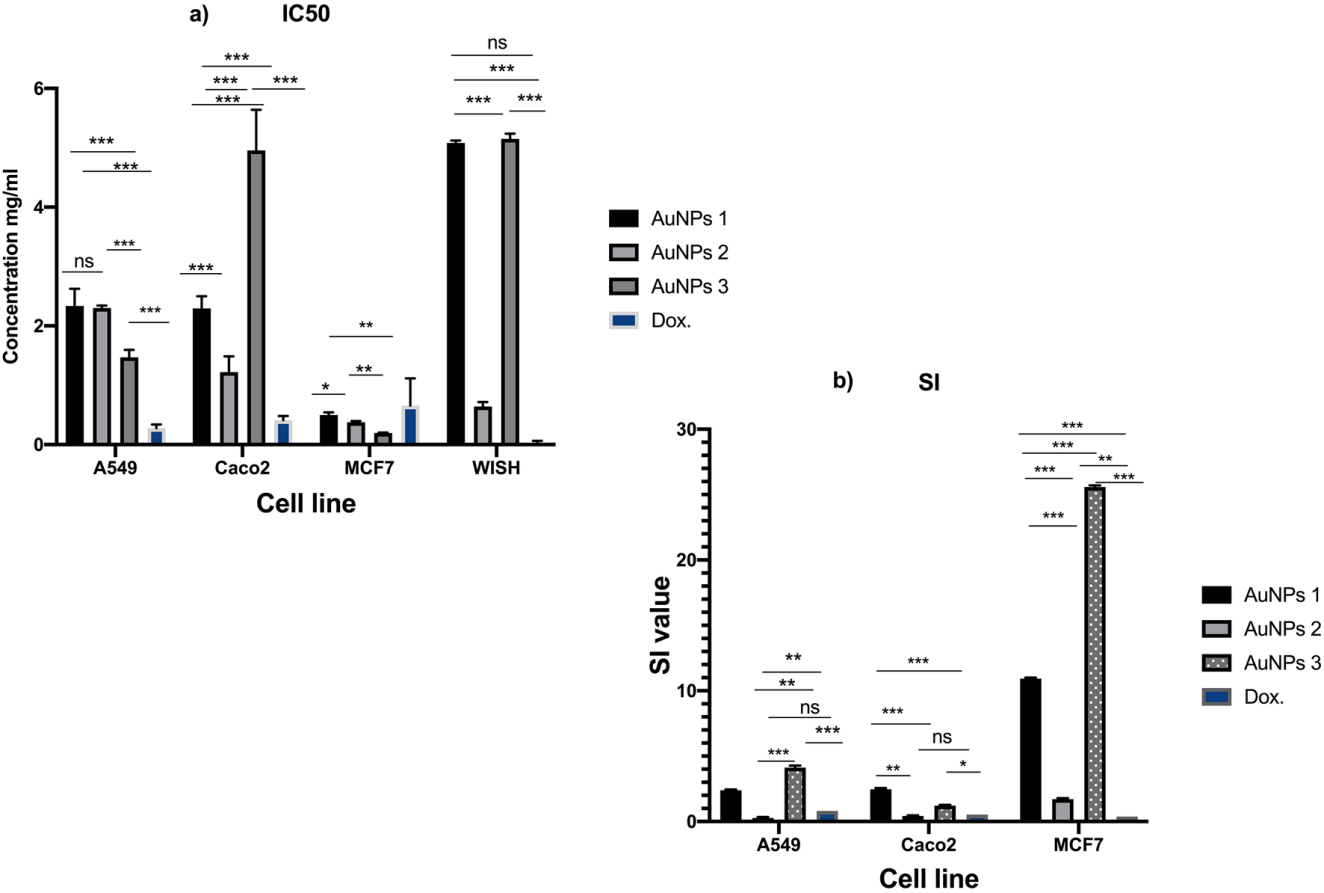
IC_50_ values and Selectivity index od AuNPs. The calculated IC50 vales of AuNPs1,2,3 were recorded on A549, CaCo-2and MCF-7 cell lines using different concentrations 2.00 mg/ml to 0.25 mg/ml (**a**). Cancer cell selectivity index of the recovered AuNPs was calculated as: SI = IC_50_nc/IC_50_cc (**b**).

**Figure 9:**
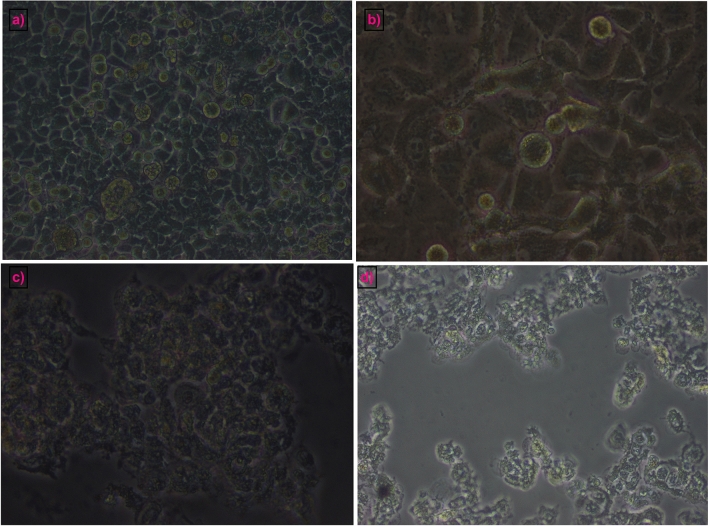
Morphological changes of MCF-7 cancer cells after AuNPs treatment. The phase contrast microscopic photos of the non-treated MCF7 cells (**a**) and the treated cells with sub IC50 dose of AuNPs1 (**b**), AuNPs2 and (**c**) AuNPs 3 (**d**).

Anticancer activities of the biogenic AuNPs were studied against A549, CaCo-2, and MCF-7 cancer cell lines upon treatment with AuNPs1, AuNPs2, and AuNPs3 sub IC_50_ concentrations. Serious morphological changes in cell structure were observed after treatment. Furthermore, the cytotoxicity results presented in Fig. 7b,c,d indicated that the biogenically-synthesized AuNPs markedly inhibited all tested cancer cell lines with different degrees. The maximum percentages of inhibition on A549 cells after AuNPs1, AuNPs2, and AuNPs3 treatments were 43.6%, 44.5%, and 41.6%, respectively (Fig. 7b**)**, and with IC_50_ values of 2.3, 2.3, and 1.4 mg/ml, respectively (Fig. 8b). Furthermore, the percentages of inhibition of AuNPs1, AuNPs2, and AuNPs3 against CaCo-2 cell line were 41.2%, 59.1%, and 14.5%, respectively (Fig. 7c) and with IC_50_ values of 2.2, 1.2, and 4.9 mg/ml, respectively (Fig. 8b). The results indicated that the most sensitive cell line to AuNPs was MCF-7 with inhibition percentages of 66.2%, 57.3%, and 70.2% after AuNPs1, AuNPs2, and AuNPs3 treatments, respectively (Fig. 7d). The calculated IC_50_ values of AuNPs on MCF-7 were 0.5, 0.37, and 0.196 mg/ml for AuNPs1, AuNPs2, and AuNPs3, respectively (Fig. 8a). It is worth to mention that the recorded IC_50_ values of AuNPs1, 2, and 3 on the non-cancerous WISH cell line were 5.08, 0.64, and 5.14 mg/ml, respectively (Fig. 8a). According to these IC_50_ values of both cancerous and non-cancerous cell lines, the selectivity index of AuNPs to cancer cells were indicated in Fig. 8b. In general, the maximum selectivity index values recorded after MCF-7 treatment with AuNPs1 and AuNPs3 were 10.37 and 25.5, respectively (Fig. 8b). Furthermore, comparing with the untreated cells (Fig. 9a), after applying the sub IC_50_ dose of AuNP1 treatment to MCF-7 (Fig. 9b), AuNPs2 (Fig. 9c), and AuNPs3 (Fig. 9d), apoptotic and dead cells occurred in major parts of the cultured plates with serious changes in cell structure and number.

#### The mode of anticancer effects of the biogenic AuNPs

##### Cell cycle analysis by flowcytometry

**Figure 10:**
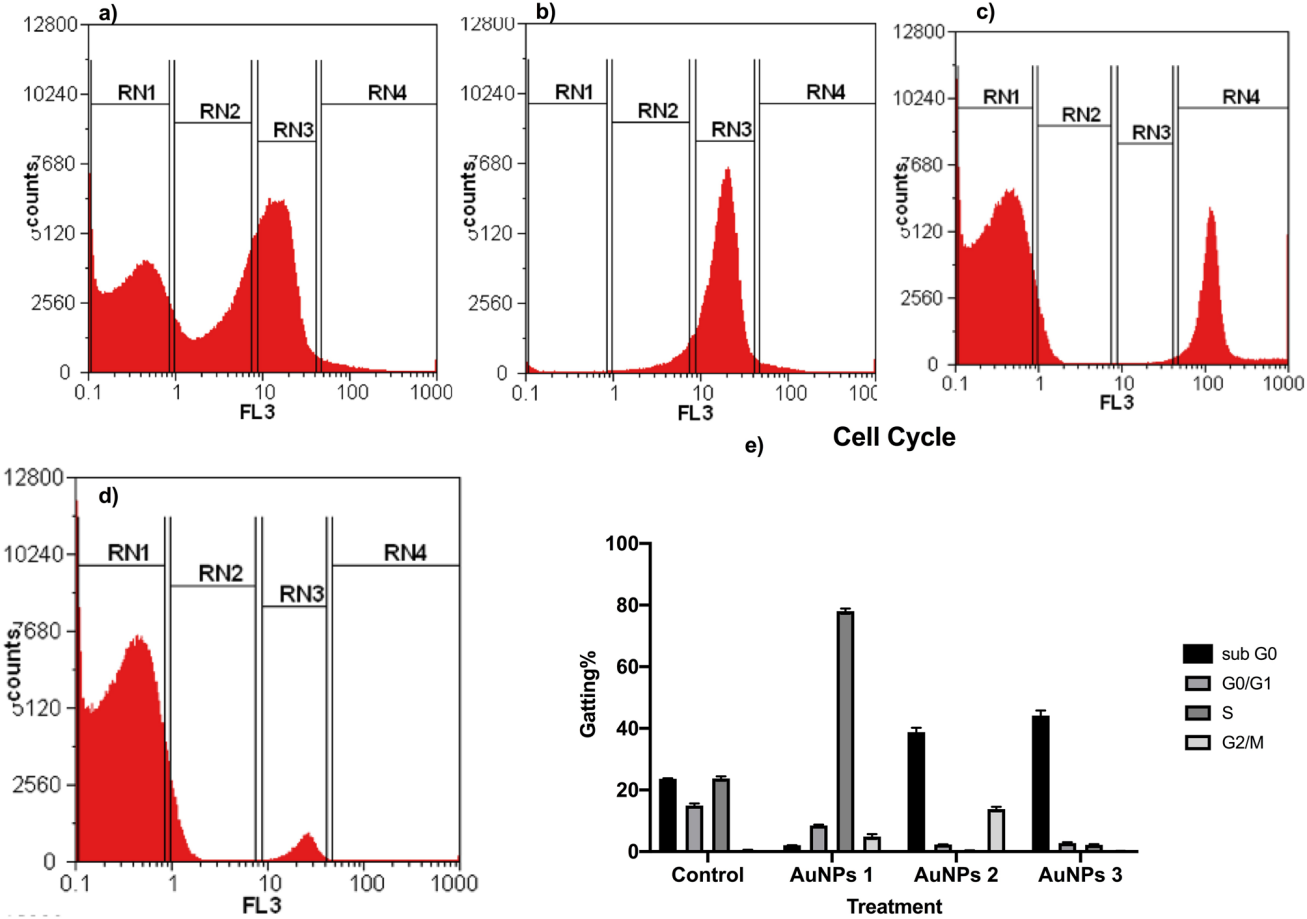
Flowcytometric analysis of MCF-7 cell lines cell cycle after AuNPs treatments. The flowcytometric analysis of MCF-7 cancer cell cycle pattern (flowcytometry charts) of the untreated cells (**a**) and MCF-7cell lines after 24 h. treatment with AuNPs1 (**b**), AuNPs2 (**c**) and AuNPs 3 (**d**). Th cell cycle patterns of untreated MCF-7 cell lines and MCF-7 cell treated with AuNPs1, AuNPs2 and AuNPs 3 after 24 h (**e**).

Flowcytometric analysis of MCF-7 cancer cell cycle pattern was performed by comparing the patterns of the treated cell with the untreated cell lines (Fig. 10). Comparing with the untreated cells (Fig. 10a), cell cycle pattern of MCF-7 cell line treatment with AuNPs1 (Fig. 10b) showed arrested cell population in S phase (77.34%), while both AuNPs2 and AuNPs3 treatments increased the cellular population in sub G0 (Apoptotic phase) with percentages of 37% ,73%, and 42.97%, respectively (Fig. 10c,d,e). Biogenic AuNPs might inhibit DNA replication and eventually arrest the MCF-7 cells cycle in S phase that demonstrated by accumulating cell populations after 24.0 h.

##### Alternation of cancer cells gene expression after MCF-7 treatment with AuNPs using RT-qPCR

**Figure 11:**
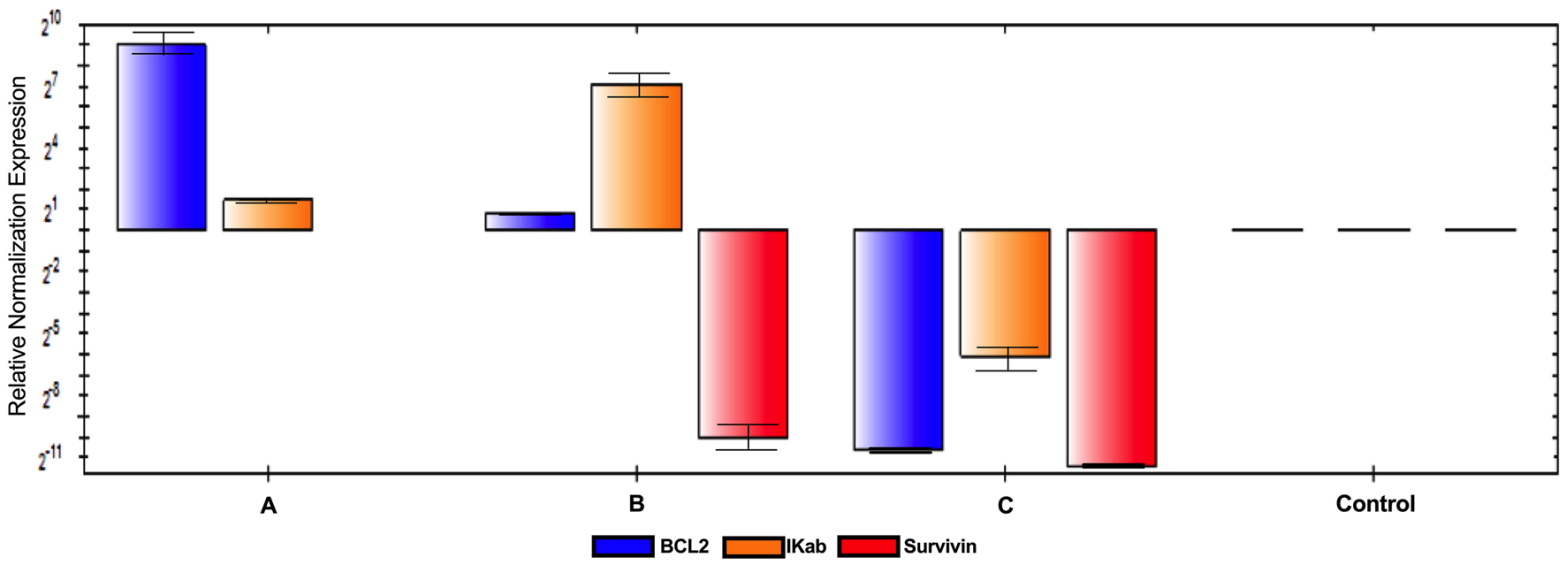
RTqPCR of MCF-7 cell lines after AuNPs treatment. The expression levels of three cancer correlated genes (Bcl2, Ikapα, and Survivn) of MCF-7 cell line treated with AuNPs1, AuNPs2 and AuNPs3 after 24 h.

MCF-7 cell line treatment with AuNPs1, AuNPs2, and AuNPs3 showed noticeable variations on the level of gene expression for the three cancer correlated genes (Bcl2, Ikapα, and Survivn) as shown in Fig. 11 and supplementary files 1–6. Firstly, AuNPs1 caused up regulation of both Bcl2 and Ikapα genes without any detected effect on Survivin expression pattern. Meanwhile, AuNPs2 up regulated both of Bcl2 and Ikapα genes and down regulated the expression of Survivin gene. Finally, AuNPs3 treatment down regulated all the gene expression levels of the three studied genes.

##### Transmission electron microscopy (TEM) of AuNPs-treated MCF-7 cell line

**Figure 12:**
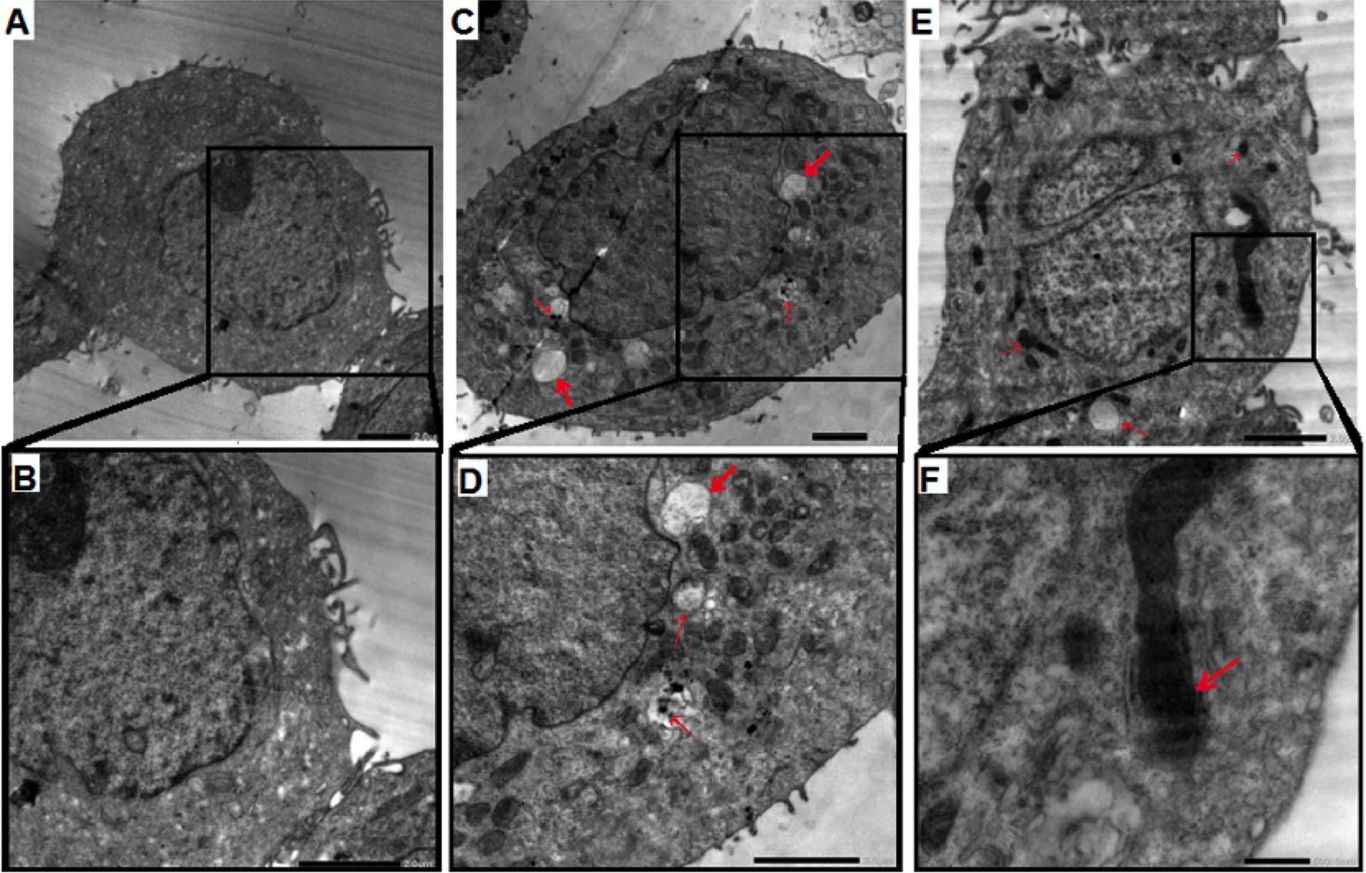
Transmission Electron Microscopy (TEM) of AuNPs-treated MCF-7 cell line. The internalization of AuNPs 3 and AuNPs2 in MCF-7 cell line after 24 h. incubation, comparing with control (untreated) cells (**a**). NPs were founded in the cytoplasm (**b**) and in vacuoles (**c**, **d**) in perinuclear region (**d**). Membrane blebbing, together with apoptotic bodies, were also noticed (**e**, **f**).

As the above mentioned results confirmed that the highest MCF-7 cellular proliferation inhibition percentage was recorded after AuNPs3 treatment. So that, we investigated the internalization of AuNPs2 and AuNPs3 in MCF-7 cell line using TEM imaging technique. Comparing with control (untreated) cells (Fig. 12a), both AuNPs were found in cell vacuoles, cytoplasm (Fig. 12b), and/or in cell perinuclear region (Fig. 12c,d), where AuNPs were taken via endocytosis (Fig. 12d). Also, some changes were recorded in the nucleus and mitochondria morphology. Furthermore, membrane blebbing together with apoptotic bodies (Fig. 12e,f) were also noticed. These results are similar to the results of flowcytometry that confirmed the induction of MCF-7 cell death after AuNPs treatment.

## Materials and methods

### Microorganisms and cell lines

Microbial strains of *Arthrospira platensis, Escherichia coli*, *Enterococcus faecalis, Candida albicans*, *Candida tropicalis*, *Salmonella enterica,* and *Streptococcus mutans* were provided by the culture collection of Genetic Engineering and Biotechnology Research Institute (GEBRI), City of Scientific Research and Technological Applications (SRTA-City), New Borg El-Arab-City, Alexandria, Egypt. All strains were isolated, purified, identified by MALDI-TOF mass spectrometry, and tested for the antimicrobial resistance in The Biopharmaceutical Products Research Department, GEBRI, SRTA-City.

Human amnion-derived normal epithelial cells ("WISH" cell line), Human, mammary gland, breast derived from metastatic site: pleural effusion adenocarcinoma cells ("MCF-7" cell line), Human colorectal adenocarcinoma, epithelial cells ("CaCo-2" cell line), and Human lung adenocarcinoma, epithelial cells ("A549" cell line) were purchased from ATCC.

### Cultivation of *Arthrospira platensis*

*Arthrospira platensis* strain was inoculated into 250.0 ml of modified Zarrouk medium, illuminated with white fluorescent light (2500 Lux) for 16.0 days at 25 ± 2 °C, and the flask was shaken twice per day to keep the culture homogenous. At the end of incubation period, the culture filtrate was collected by centrifugation at 4000 rpm for 30.0 min at 4.0 °C and the recovered filtrate was further purified using a Whatman filter paper to remove any remaining suspended algal biomass^39^.

### Biosynthesis of gold nanoparticles

#### Extraction of water soluble polysaccharides

The total cellular metabolites of *Arthrospira platensis* were collected from the culture as previously described^39^. Briefly, an aliquot of 500 ml of algal filtrate was boiled for 30 min at 100 °C and allowed to cool down at room temperature. Polysaccharides were precipitated by the addition of fourfold ethyl alcohol 95.0% (v/v). The precipitated polysaccharides were collected by filtration using Whatman filter papers (110 mm) and washed by absolute ethanol and left to dry in oven at 50 °C to complete dryness.

#### Biosynthesis of AuNPs by thermal reduction method

Sodium tetrachloroaurate (III) dihydrate (NaAuCl_4_·2H_2_O, Sigma-Aldrich) was used to prepare a stock solution of initial concenration 1.0 mM in ultra-high purified water (Milli-Q plus system, Millipore Co., USA). The prepared solution was boiled for 30.0 min then left to cool down at room temperature. This pre-boiled stock solution was used to prepare AuNPs using two different methods; thermal reduction and l-ascorbic acid reduction methods.

The following experiments were performed using different dilutions of the NaAuCl_4_ stock solutions (0.25, 0.50, and 1.00 mM) to adjust the optimum molar ratios used in the biosynthesis of AuNPs. The best NaAuCl_4_ concentration was used in further optimization processes to maximize the production of AuNPs as follows.

In the first experiment; (NaAuCl_4_:polysaccharides (1:1 molar ratio), 100.0 ml of 0.25 mM pre-boiled NaAuCl_4_ solution (optimum concentration) was added to 100.0 ml of 0.5% EPS solution (w/v) (pH = 10.2 at 20.0 °C) in a 500.0 ml round-bottom flask, where this polysaccharide solution was previously pre-boiled with vigorous stirring for 1.0 h. After that, the mixture was left to boil for 30.0 min until the formation of intense ruby red color. This colloidal solution was labeled as AuNPs1.

In the second experiment; NaAuCl_4_:polysaccharides (2:1 molar ratio), 200.0 ml of 0.25 mM pre-boiled NaAuCl_4_ solution was added to 100.0 ml of 0.5% polysaccharide solution (w/v) (pH = 10.2 at 20.0 °C) in a 500.0 ml round-bottom flask, and the same procedure was repeated as described above. The generated colloidal solution with purple color was labeled as AuNPs2.

#### Biosynthesis of AuNPs by l-ascorbic acid reduction method

A volume of 100.0 ml of 0.5% EPS solution (w/v) (pH = 10.2 at 20.0 °C) was added into a 500.0 ml beaker and stirred vigorously at room temperature, then 100.0 ml of the previously pre-boiled NaAuCl_4_ solution was added to it. After that, 5.0 ml of 0.35% l-ascorbic acid (Sigma-Aldrich) solution (w/v) was added drop-wise with continuous stirring until the instant formation of dark violet color. The colloidal solution with dark violet color was labeled as AuNPs3.

### Characterization of the synthesized AuNPs

#### UV–Vis spectral analysis

UV–Vis spectroscopic measurements were performed for 3.0 ml of the produced homogenous colloidal solutions of each AuNPs preparation. The measurements were carried out on a T80 + spectrophotometer (PG instruments, T60 UV–Vis Spectrophotometer United Kingdom) at wave lengths ranged between 400.0 and 1100.0 nm using a quartz cuvette of 10.0 mm path length. The samples were scanned at the central laboratory of Faculty of Science, Alexandria University, Alexandria, Egypt.

#### X-ray powdered diffraction (XRD) analysis

The obtained AuNPs preparations were dried out at 50.0 °C for 16.0 h and the purified dried powders were scanned using diffraction angles (2Ɵ) that ranged between 5.0° and 80.0°. The XRD patterns of the produced nanoparticles were measured using a diffractometer (LabX-6100, SHIMADZU) with a 40-kV voltage and a 30.0 mA electric current employing Cu Kα radiation (λ = 1.5418 Å). The analysis was performed at the Egypt-Japan University of Science and Technology (E-JUST).

Nanoparticles sizes were estimated using XRD measurements by applying Debye Scherrer equation, where strong reflections with large intensities were used to measure the full width at half maximum (FWHM). The Scherrer equation for calculating the particle size is given by:

$${\mathrm{D}}=\frac{K\uplambda }{{\upbeta \cos \uptheta }}$$

where D = the crystallite size in the direction perpendicular to the lattice plane, K = Scherrer constant "crystal shape factor", λ = wavelength of light used for the diffraction, β = full width at half maximum (FWHM), and θ = Bragg angle.

The Scherrer constant (K) in the above formula accounts for the shape of the particle and is generally taken to have the value 0.9 as a good approximation.

#### Transmission electron microscopy (TEM)

AuNPs samples were collected by centrifugation at 9000.0 rpm for 30.0 min. The pellets were dispersed in double distilled water and sonicated to remove any clumps. Structural characterization of the generated biogenic AuNPs was carried out by a JEM-2100F Transmission Electron Microscope (JEOL, Japan) at E-JUST with 200.0 kV magnification power. The samples for TEM characterization were prepared by placing a 5.0 μl of the colloid solution on a carbon coated 3 mm copper grid, until the formation of a thin film of the sample on the grid, and then the grid was left to dry at room temperature**.**

#### FTIR analysis

The dried AuNPs were analyzed using IRTracer-100 spectrophotometer (SHIMADZU) at the central laboratory of SRTACity.

### Biological activities of AuNPs

#### Antimicrobial activities of AuNPs

According to the Clinical and Laboratory Standards guidelines (CLSI) with minor modifications, different concentrations of AuNPs were prepared and 100.0 µl of each concentration was incubated with about 10^6^ CFU/ml of each of the following antimicrobial resistant microbial strains (*Escherichia coli*, *Enterococcus faecalis, Candida albicans*, *Candida tropicalis*, *Salmonella enterica,* and *Streptococcus mutans)* in a 96 well plate. In addition, 100.0 µl of LB media was added to 100.0 µl of the microbial culture to setup the experimental control groups. The inoculated plates were incubated at 37.0 °C for 24.0 h. After incubation, the absorbance was measured at 620.0 nm using automated ELIZA microplate reader BINDER BIOTECK E LX 800 (Germany)^40^. The inhibition percentage of AuNPs was calculated according to the following equation:

$$ {\text{Inhibition}}\;{\text{percentage}} = \left( {{\text{A}} - {\text{A1}}/{\text{A}}0} \right) \times 100 $$

where A: the absorbance of the treatment group, A1: the absorbance of the blank, and A0: the absorbance of the control group.

The lowest concentration of the tested extracts resulted in the inhibition of bacterial growth was taken as the Minimal Inhibitory Concentration (MIC).

#### Safety assay and anticancer activities of AuNPs

##### Mammalian cell lines culture

WISH, CaCo-2, and A549 cell lines were cultured on DMEM media (Sigma-Aldrich), while MCF-7 cell line was cultured on RBMI media. The used media were supplemented with 200.0 mM l-glutamine (Lonza) and 10.0% fetal bovine serum (Bio Whittaker) and 1.0% penicillin/streptomycin. Cells were seeded in 25.0 cm tissue culture flasks and incubated for 24.0 h or till confluency at 37.0 °C in a 5.0% CO_2_ humidified incubator.

##### Safety assay of AuNPs

The safety assay was performed to determine the concentration that does not depict any toxic effects on the non-cancerous cell line (WISH cells)^41^. In 96-well plates, 100.0 µl of 6 × 10^4^ cell/ml cells was seeded in each well and the plates were incubated at 37.0 °C in a humidified 5.0% CO_2_ incubator for 24.0 h. After incubation, the exhausted old medium was replaced with 100.0 µl of different treatment concentrations (previously prepared in RPMI medium starting from 2.0 to 0.25 mg/ml). The treated plates were incubated at the same growth conditions for 24.0 h. At the end of incubation, cellular viability was quantified using MTS assay kit (Promega, USA) according to the manual. Briefly, MTS Reagent Powder is a novel tetrazolium compound [3-(4,5-dimethylthiazol-2-yl)-5-(3-carboxymethoxyphenyl)-2-(4-sulfophenyl)-2H-tetrazolium, inner salt] and an electron coupling reagent (phenazine ethosulfate; PES). PES has enhanced chemical stability, which allows it to be combined with MTS to form a stable solution. About 20.0 µl of the solution was directly added to each well at the end of incubation with the treatment and then the plates were incubated for 2.0–4.0 h. The quantity of formazan products was quantified at 490.0 nm, which indicates the number of living cells.

$$ {\text{Cytotoxicity}}\;{\text{percentage}} = \left( {{\text{A}} - {\text{A1}}/{\text{A}}0} \right) \times 100 $$

where A: the absorbance of the treatment group, A1: the absorbance of the blank, and A0: the absorbance of the control group.

##### In vitro anticancer activity of green synthesized NPs

Anticancer activities of AuNPs against A549, CaCo-2, and MCF-7 cell lines were investigated using MTS assay protocol as described in the safety assay method. The morphological changes occurred in cancer cells, post AuNPs treatment, were inspected using CKX41 Olympus Inverted Microscope, Japan.

##### Selectivity index (SI)

Cancer cell selectivity index of the biogenic AuNPs was calculated as reported by Koch et al.^42^ with a minor modification;

$$ {\text{SI}} = {\text{IC}}_{{{5}0}} {\text{nc}}/{\text{IC}}_{{{5}0}} {\text{cc}} $$

where IC_50_nc refers to the IC_50_ value of the tested compound on normal cells, IC_50_cc refers to the IC_50_ of the tested compound on the tested cancer cell line.

#### Mode of anticancer action of AuNPs treatments against A549, CaCo-2, and MCF-7 cell lines

##### Cell cycle analysis by flowcytometry

The alterations in cell cycle patterns were checked using propidium iodide (PI) method and flowcytometer (BD FACSCanto, USA)^43,44^. After 48.0 h of AuNPs-cellular treatment, the collected cells (approximately 2.0 × 10^5^ cells/ml) were re-suspended in warm PBS, fixed with 4.0 ml ice cold absolute ethanol (drop-wisely added), and then stained for 30.0 min with 0.5 ml of warm PI working solution (0.35 ml of PI stock solution (1.0 mg/ml) and 0.7 ml RNase A solution (1.0 mg/ml)). All samples were kept under 4.0 °C until flowcytometric measurement.

##### Gene expression pattern alternation in MCF-7 cancer cell line after AuNPs treatments

The anticancer molecular mode of action of AuNPs was studied via screening their activities in controlling the expression of Bcl2, IKap-α, and Survivin genes (Table 3) in MCF-7 cells (the most sensitive cancer cell line for AuNPs). After cellular treatment, MCF-7 cell line was cultured in 12.0-well plates (6.0 × 10^3^ cell/ml) for 24.0 h along with the resulted non-toxic concentration of AuNPs. After incubation, total cellular RNA was extracted using RNA extraction kit (Qiagen). Then, cDNA was synthesized using Oligo-dT primer and AMV reverse transcriptase (Promega Corp., Madison, WI). β-actin primers were used to amplify the house-keeping, β-actin, gene as an internal control for standardization of PCR products. The RTq-PCR was done using SYBR Green dye (QuantiTect SYBR Green PCR Kits) and Light Cycler fluorimeter Bio-Rad S1000 Tm thermal cycler (Bio-Rad, USA).

**Table 3:** Primers sequence used for quantitative real time PCR.

Gene	Sequences
Bcl2	F: 5′-TATAAGCTGTCGCAGAGGGGCTA-3′
	R: 5′-GTACTCAGTCATCCACAGGGCGAT-3′
IKap-α	F: 5′-CATGAAGAGAAGACACTGACCATGGAAA-3′
	R: 5′-TGGATAGAGGCTAAGTGTAGACACG-3′
Survivin	F: 5′-TGCCCCGACGTTGCC-3′
	R: 5′-CAGTTCTTGAATGTAGAGATGCGGT-3′
*GAPDH*	F: 5′-TGCCCTCAACGACCACTTTG-3′
	R: 5′-TACTCCTTGGAGGCCATGTG-3′

##### Transmission electron microscopy for MCF-7 treated cells

The most sensitive cell line to AuNPs treatments was selected to be scanned using Transmission Electron Microscope (JEOL, Japan). The treated MCF-7 cells were collected, fixed, and dehydrated using a series of acetone washes. Then, the cells were passed through a transition solvent such as propylene oxide, infiltrated, and finally embedded in a liquid resin (epoxy and LR White resin). After embedding, the solidified resin block was then sectioned by ultramicrotome, where sections of 50.0–70.0 nm thickness were made using a diamond knife. The sections were mounted on TEM grids and stained with 4.0% Uranyl acetate for 25.0 min. Then, the grids were rinsed four times with pure water before staining with 1.0% lead citrate for 5.0 min and rinsing with pure water. The grids were finally stored in a grid box until examination under TEM.

### Statistical analysis

Data are presented as mean ± SD. Two means were compared by Student’s *t-test* and three or more group were meant by one-way analysis of variance (ANOVA) with X tests for pair-wise comparisons. A *p* value < 0.05 was considered significant. All statistical calculations were conducted using Graph Pad Prism 7 software.

## Discussion

Nanoparticles and nanotechnology are playing important roles in different fields such as medicine, biology, physics, chemistry, and sensing due to their distinctive properties^45^. The nanoparticles of noble metals (Cu, Hg, Ag, Pt, and Au) comparing with other metal nanoparticles, are increasingly attracting researchers attention^45^ due to their unique optical and electrical properties. For example, AuNPs was used in various applications of interdisciplinary branches of science including medicine, material science, biology, chemistry and physics^46^. The FDA organization has approved the conjugation of anticancer drugs, diagnostic agents, and/or targeting agents to nanobiomaterials to build nanostructure weapons against cancer cells^2^. At the time being, AuNPs are prepared by different green and synthetic techniques with different shapes as nanospheres, nanorods, nanocubes, nanobranches, nanobipyramids, nanoflowers, nanoshells, nanowires, and nanocages^47,48^. Green chemistry is one of the promising research areas in nanotechnology for the fabrication of nanomaterials due to the growing demand to the synthesis of environmentally safe nanomaterials^49^ and to reduce the cost and energy consumption associated with the production process using the physical/chemical techniques^50^. The green synthesis approach are nontoxic, one step, easily available in affordable value, and thus are more preferable than the chemical and physical approaches^8^. Metallic nanoparticles have been biosynthesized using various natural resources as algae^39,51^, fungi^52–54^, bacteria^55^, and plants^56,57^ as reducing and stabilizing agents for the fabrication of nanoparticles with different morphologies^58^ and size distributions.

Therefore, in this study, we reported for the first time the green synthesis of biologically effective AuNPs via different concentrations of *Arthospira platenisis* exopolysaccharides and evaluated their efficacy against various cancer cell lines and microbial strains. The majority of the published research studies reported plant-mediated synthesis processes for the production of AuNPs and the most effective biogenic particles were spherical or nearly spherical shaped AuNPs with diameter size less than 100.0 nm^59^. Our recovered biogenic AuNPs2 showed a range of particle sizes from 8.0 to 40.0 nm, the particles were isotropic, which are well known for small spherical nanoparticles due to their small aspect ratio^60,61^. Each of the prepared AuNPs colloidal solutions expressed a very intense color, which is not found in the case of its parent material as previously reported in the published literatures^62–64^. The reason behind these colors is attributed to the collective oscillation of free conduction electrons. The surface plasmon resonance (SPR) absorbance is highly sensitive to the size, nature, temperature, shape of particles, and the environment of the surroundings^65^. Generally, gold nanoparticles show intensive SPR bands in the region of 500.0 to 600.0 nm at the visible spectral region depending on the method of fabrication, the size of the particles, and the surrounding parameters^66,67^. Also, UV–Vis spectrum of the recovered AuNPs showed absorption band at 560.0 nm, which indicate a little red shift as a result of the occurrence of some degree of aggregation. The aggregation states could be detected visually by a change in color of the solution from red to blue or purple. Blue shift was observed in the current study from 550.0 to 530.0, which emphasis the dependence of spectra on the size of AuNPs, as the size of gold nanoparticles decreases, the maximum absorption peaks were shifted to smaller wavelengths, a phenomenon called "Blue Shift"^68^. Different research studies showed that different biological sources which were used for AuNPs biosynthesis significantly affected their biological activities. As the biomolecules extracted from natural sources are used as unique reducing and capping agents for the reduction of metallic ions into differently-shaped and effective nanoparticles, whose specific chemistry are definitely linked to the efficacies of the parent materials^69^. In addition to the type of the capping agents used in NPs preparation, nanoparticles characteristics such as their shape, size, surface chemistry, and charge could affect their pharmacokinetics (absorption, metabolism, distribution, and elimination)^70^. These findings could explain the cytotoxic effects of the prepared AuNPs in the current study, as it was reported that AuNPs with average size < 30.0 mm were more cytotoxic and could be endocytosed by cells^71^. Furthermore, capping agents and NP characteristics such as shape, size, surface chemistry, and charge also influence the safety properties of NPs. Although different research articles confirmed the significant biomedical activities of biogenic metallic nanoparticles, it is very important to detect the hazards associated with the use of biogenic NPs. It is noteworthy that AuNPs safety is a hot topic that has gained great interest due to their potential biotechnological applications, but their safety is still a matter of debate among scientists. A large number of in vitro and in vivo experiments have proved the safety of AuNPs, while others confirmed their toxicity^70^. Different factors such as size, shape, surface capping materials, charges of the surface, dose, and exposure time affect the toxicological properties of the prepared nano-particles^72^. In the current study, our results indicated that the safest concentration of AuNPs on WISH cells was 30.0 μg/ml), which is a very low concentration comparing with both the reported concentrations in the scientific literature and the previously reported concentration of the biologically prepared AgNPs using the same polysaccharides^39^. In addition, another report explained that the biogenic AuNPs exert their toxic effect at concentrations as low as 40.0 μg/ml^73^.

The antibacterial effects of nanomaterials such as silver, gold, copper, titanium, zinc oxide, and magnesium oxide made them potential substitutes or complementary agents for antibacterial therapies^74^. However, there is no clear explanation in the scientific literature on the exact mechanism of NPs antibacterial effects, it is hypothetical that NPs could target the microbial cell membranes and damage the membrane potential. By considering the biomolecules capping of the prepared NPs surface, we believe that their strong antibacterial effects could be related to an easier penetration of the cell membranes by these biomolecules that resulted in increasing toxicity. Our results indicated that Gram negative bacteria were more sensitive to AuNPs treatments and this could be the result of the presence of tough cell wall in Gram-positive bacteria, but gram-negative bacterial cell wall is thinner. Therefore, AuNPs easily penetrate the cell membrane of the gram-negative bacteria and cause damaging effects to the bacterial cell^75^. This finding confirmed the antimicrobial effects of AuNPs that could be used as a potential antimicrobial agent in the future.

The different anticancer effects of the biologically synthesized NPs on the same cell lines was found to be related to the nature of the biological capping agents, the metal NPs size distribution and shapes, and^76^ the experimental conditions such as pH, temperature, and the concentration of metal salt^8,76,77^. As we mentioned above, the different cytotoxic effects of nanoparticles could be attributed to the different nature of the capping agent present in algal polysaccharides. Although, the variable cytotoxic effects of the biogenic nanoparticles on different mammalian cells could be attributed to the size and level of aggregation of the NPs^72^. Various bio-molecular agents were reported to be involved in AuNPs synthesis and stabilization into smaller nanoclusters. These agents could be amino acids, protein side chains, glutathione, phospholipids, and many more agents^72^.

The results of screening the efficacy of the biogenic AuNPs against breast cancer could be summarized in 4 groups^59^, the first group confirmed the cytotoxic effects of biogenic AuNPs against breast cancer cells, while the second one reported no cytotoxic effects were observed upon cancer cells treatment with the nanostructures. In addition, the third group confirmed significant cytotoxic effects of the biogenic AuNPs against breast cancer cells, but low or no cytotoxicity against non-cancerous cells. Meanwhile, the last group didn’t record any cytotoxic effects against both cancerous and non-cancerous cells. Interestingly, no reports were found to confirm the higher cytotoxic effects of the biogenic AuNPs on non-cancerous cells over breast cancer cells^59^. In agreement with the third group, the current study confirmed the anticancer selectively effects of the biogenic AuNPs against MCF-7 cell line with low cytotoxic effects against noncancerous cells (about 25 times cytotoxic effects on the MCF-7 cells over the non-cancerous cells). The anticancer effects of the biogenic AuNPs were explained by arresting MCF-7 cells in the S phase and increasing the cellular population in the sub G0 phase. Similar results were found by earlier reports on MCF-7 and S phase arrest in MDA-MB-231^78–80^. Furthermore, decreasing the size of materials to the nanoscale extraordinary increases the reactivity and subsequently the interaction of NPs with the biological entities. Our recovered AuNPs were found to be deposited in the vacuoles, the cytoplasm, and in the perinuclear region of MCF-7 cells. AuNPs may be internalized into these cellular structures and caused different ultrastructural modifications. Generally, positively charged molecules have higher uptake ratio, but poor intracellular stability comparing to neutral or negatively charged molecules^81^. Furthermore, nanoparticles size positively affect their internalization^82^. Two main AuNPs internalization mechanisms were reported: membrane-bound vesicles and endosomes^83^. It has been indicated that AuNPs rods may be endosomes internalized by vesicular bodies into human dermal fibroblasts and colon adenocarcinoma^84,85^. Other studies reported that AuNPs could be phagocytic internalized in A549 and HBL-100 cells^86,87^. Furthermore, AuNPs can be located in the cytosol, lysosomes, and perinuclear region as aggregates or singletons^86,87^. The abilities of biogenic nanoparticles to alter the expression patterns of cancer cell genes and their genotoxic effects in different in vitro and in vivo models were reported^51,88^. Furthermore, the abilities of AuNPs to induce cellular apoptosis by decreasing the expression levels of Survivin and Bcl-2 were confirmed. Choudhury et al., reported a reduction in the level of Bcl-2 protein (anti-apoptotic protein) in A549 cells after treatment with 40 nm AuNPs^89^. Also, Selim et al., indicated that AuNPs could decreased the level of Bcl-2 proteins in the treated MCF-7 cells^90^.

## Conclusion

In the present work, it has been demonstrated that *Arthospira platensis* exopolysaccharides (EPS) are capable of reducing gold ions into three different AuNPs and the generated nanoparticles were stable for more than 3 months. The biosynthesized nanoparticles showed potent antimicrobial and cytotoxic effects against the tested cell lines and microbial strains. This study opens the door for the usage of the biogenic AuNPs alone or in parallel with chemotherapies and antibiotics for future cancer and microbial therapies, respectively.

## Future prospective

Although we provided strong and comprehensive in vitro results, further biological applications and in vivo studies are required to confirm the reliability and efficacy of these AuNPs in animal models, which is our future prospective.

## Supplementary Information

Supplementary Information 1.

Supplementary Information 2.

Supplementary Information 3.

Supplementary Information 4.

Supplementary Information 5.

Supplementary Information 6.

## Data Availability

All data generated or analyzed during this study are included in this published article [and its supplementary information files].

## References

[1] Aghamiri S, Jafarpour A, Malekshahi ZV, Mahmoudi Gomari M, Negahdari B (2019). Targeting siRNA in colorectal cancer therapy: Nanotechnology comes into view. J. Cell. Physiol..

[2] Saravanan, M. *et al.* in *Handbook on Nanobiomaterials for Therapeutics and Diagnostic Applications* (eds Krishnan, A. *et al.*) 439–456 (Elsevier, 2021).

[3] Jin R, Cao YJS (2001). CA; Mirkin, KL Kelly, GC Schatz and JG Zheng. Science.

[4] Alivisatos AP (1996). Perspectives on the physical chemistry of semiconductor nanocrystals. J. Phys. Chem..

[5] Aizpurua J (2003). Optical properties of gold nanorings. Phys. Rev. Lett..

[6] Seo D (2008). Shape adjustment between multiply twinned and single-crystalline polyhedral gold nanocrystals: Decahedra, icosahedra, and truncated tetrahedra. J. Phys. Chem..

[7] Jain PK, Lee KS, El-Sayed IH, El-Sayed MA (2006). Calculated absorption and scattering properties of gold nanoparticles of different size, shape, and composition: Applications in biological imaging and biomedicine. J. Phys. Chem. B.

[8] Barabadi H (2019). Emerging theranostic silver and gold nanomaterials to combat prostate cancer: A systematic review. J. Cluster Sci..

[9] Barabadi H (2020). Emerging theranostic gold nanomaterials to combat colorectal cancer: A systematic review. J. Cluster Sci..

[10] Boomi P (2020). Phyto-engineered gold nanoparticles (AuNPs) with potential antibacterial, antioxidant, and wound healing activities under in vitro and in vivo conditions. Int. J. Nanomed..

[11] Mohamed MM, Fouad SA, Elshoky HA, Mohammed GM, Salaheldin TA (2017). Antibacterial effect of gold nanoparticles against *Corynebacterium pseudotuberculosis*. Int. J. Vet. Sci. Med..

[12] Fuster MG (2020). Antibacterial effect of chitosan–gold nanoparticles and computational modeling of the interaction between chitosan and a lipid bilayer model. Nanomaterials.

[13] Barabadi H (2019). A systematic review of the genotoxicity and antigenotoxicity of biologically synthesized metallic nanomaterials: Are green nanoparticles safe enough for clinical marketing?. Medicina.

[14] Ankamwar B, Chaudhary M, Sastry M (2005). Gold nanotriangles biologically synthesized using tamarind leaf extract and potential application in vapor sensing. Synth. React. Inorg. Metal Organ. Nano-Metal Chem..

[15] Kumar V, Yadav SC, Yadav SK (2010). *Syzygium cumini* leaf and seed extract mediated biosynthesis of silver nanoparticles and their characterization. J. Chem. Technol. Biotechnol..

[16] Tripathy A, Raichur AM, Chandrasekaran N, Prathna T, Mukherjee A (2010). Process variables in biomimetic synthesis of silver nanoparticles by aqueous extract of *Azadirachta indica* (Neem) leaves. J. Nanopart. Res..

[17] Leela A, Vivekanandan M (2008). Tapping the unexploited plant resources for the synthesis of silver nanoparticles. Afr. J. Biotechnol..

[18] Mohanpuria P, Rana NK, Yadav SK (2008). Biosynthesis of nanoparticles: Technological concepts and future applications. J. Nanopart. Res..

[19] Chandran SP, Chaudhary M, Pasricha R, Ahmad A, Sastry M (2006). Synthesis of gold nanotriangles and silver nanoparticles using Aloevera plant extract. Biotechnol. Prog..

[20] Mehra RK, Winge DR (1991). Metal ion resistance in fungi: Molecular mechanisms and their regulated expression. J. Cell. Biochem..

[21] Sawle BD (2008). Biosynthesis and stabilization of Au and Au–Ag alloy nanoparticles by fungus, *Fusarium semitectum*. Sci. Technol. Adv. Mater..

[22] He S (2007). Biosynthesis of gold nanoparticles using the bacteria *Rhodopseudomonas capsulata*. Mater. Lett..

[23] Huang J (2007). Biosynthesis of silver and gold nanoparticles by novel sundried *Cinnamomum camphora* leaf. Nanotechnology.

[24] Gardea-Torresdey J (2002). Formation and growth of Au nanoparticles inside live alfalfa plants. Nano Lett..

[25] Shankar SS (2004). Biological synthesis of triangular gold nanoprisms. Nat. Mater..

[26] Shankar SS, Ahmad A, Sastry M (2003). Geranium leaf assisted biosynthesis of silver nanoparticles. Biotechnol. Prog..

[27] Oliveira EG, Rosa GS, Moraes MA, Pinto LA (2009). Characterization of thin layer drying of *Spirulina platensis* utilizing perpendicular air flow. Bioresour. Technol..

[28] Belay A, Gershwin M (2007). Spirulina in Human Nutrition and Health.

[29] Choopani A (2016). A review on antioxidant properties of Spirulina. J. Appl. Biotechnol. Rep..

[30] Ciferri O (1983). Spirulina, the edible microorganism. Microbiol. Rev..

[31] Doshi H, Ray A, Kothari IJ (2007). Bioremediation potential of live and dead Spirulina: Spectroscopic, kinetics and SEM studies. Biotechnol. Bioeng..

[32] Liu Q, Huang Y, Zhang R, Cai T, Cai Y (2016). Medical application of spirulina platensis derived C-phycocyanin. Evid. Based Complement. Altern. Med. eCAM.

[33] Lai F, Wen Q, Li L, Wu H, Li X (2010). Antioxidant activities of water-soluble polysaccharide extracted from mung bean (*Vigna radiata* L.) hull with ultrasonic assisted treatment. Carbohydr. Polym..

[34] Chaiklahan R (2013). Polysaccharide extraction from *Spirulina* sp. and its antioxidant capacity. Int. J. Biol. Macromol..

[35] Capek P, Machová E, Turjan J (2009). Scavenging and antioxidant activities of immunomodulating polysaccharides isolated from *Salvia officinalis* L. Int. J. Biol. Macromol..

[36] Klaus A (2011). Antioxidative activities and chemical characterization of polysaccharides extracted from the basidiomycete *Schizophyllum commune*. LWT Food Sci. Technol..

[37] Balachandran P, Pugh ND, Ma G, Pasco DS (2006). Toll-like receptor 2-dependent activation of monocytes by *Spirulina polysaccharide* and its immune enhancing action in mice. Int. Immunopharmacol..

[38] Sathiyanarayanan G, Kiran GS, Selvin J (2013). Synthesis of silver nanoparticles by polysaccharide bioflocculant produced from marine *Bacillus subtilis* MSBN17. Colloids Surf. B Biointerfaces.

[39] El-Deeb NM (2020). Biogenically synthesized polysaccharides-capped silver nanoparticles: Immunomodulatory and antibacterial potentialities against resistant *Pseudomonas aeruginosa*. Front. Bioeng. Biotechnol..

[40] Bonacorsi C, Raddi MSG, Carlos IZ, Sannomiya M, Vilegas W (2009). Anti-*Helicobacter pylori* activity and immunostimulatory effect of extracts from *Byrsonima crassa* Nied. (Malpighiaceae). BMC Complement. Altern. Med..

[41] Borenfreund E, Puerner JA (1985). Toxicity determined in vitro by morphological alterations and neutral red absorption. Toxicol. Lett..

[42] Koch A, Tamez P, Pezzuto J, Soejarto D (2005). Evaluation of plants used for antimalarial treatment by the Maasai of Kenya. J. Ethnopharmacol..

[43] Li Y (2001). Novel antitumor artemisinin derivatives targeting G1 phase of the cell cycle. Bioorg. Med. Chem. Lett..

[44] Leonce S (2001). Induction of cyclin E and inhibition of DNA synthesis by the novel acronycine derivative S23906–1 precede the irreversible arrest of tumor cells in S phase leading to apoptosis. Mol. Pharmacol..

[45] Ramalingam V (2014). Biosynthesis of silver nanoparticles from deep sea bacterium *Pseudomonas aeruginosa* JQ989348 for antimicrobial, antibiofilm, and cytotoxic activity. J. Basic Microbiol..

[46] Khanna P, Kaur A, Goyal D (2019). Algae-based metallic nanoparticles: Synthesis, characterization and applications. J. Microbiol. Methods.

[47] O'Neal DP, Hirsch LR, Halas NJ, Payne JD, West JL (2004). Photo-thermal tumor ablation in mice using near infrared-absorbing nanoparticles. Cancer Lett..

[48] Xiao T, Huang J, Wang D, Meng T, Yang X (2020). Au and Au-based nanomaterials: Synthesis and recent progress in electrochemical sensor applications. Talanta.

[49] Naghdi M (2015). Green and energy-efficient methods for the production of metallic nanoparticles. Beilstein J. Nanotechnol..

[50] Duan H, Wang D, Li Y (2015). Green chemistry for nanoparticle synthesis. Chem. Soc. Rev..

[51] El-Deeb NM, Abo-Eleneen MA, Awad OA, Abo-Shady AM (2022). Arthrospira platensis-mediated green biosynthesis of silver nano-particles as breast cancer controlling agent. In vitro and in vivo safety approaches. Appl. Biochem. Biotechnol..

[52] Golnaraghi Ghomi AR (2019). Fungus-mediated extracellular biosynthesis and characterization of zirconium nanoparticles using standard Penicillium species and their preliminary bactericidal potential: A novel biological approach to nanoparticle synthesis. Iran. J. Pharmaceut. Res..

[53] Honary S, Barabadi H, Ebrahimi P, Naghibi F, Alizadeh A (2015). Development and optimization of biometal nanoparticles by using mathematical methodology: A microbial approach. JNanoR.

[54] Barabadi H, Kobarfard F, Vahidi H (2018). Biosynthesis and characterization of biogenic tellurium nanoparticles by using *Penicillium chrysogenum* PTCC 5031: A novel approach in gold biotechnology. Iran. J. Pharm. Res..

[55] Nair B, Pradeep T (2002). Coalescence of nanoclusters and formation of submicron crystallites assisted by *Lactobacillus* strains. Cryst. Growth Des..

[56] Al-Zahrani SA (2022). Anticancer potential of biogenic silver nanoparticles using the stem extract of *Commiphora gileadensis* against human colon cancer cells. Green Process. Synth..

[57] Yassin AM (2017). Induction of apoptosis in human cancer cells through extrinsic and intrinsic pathways by *Balanites aegyptiaca* furostanol saponins and saponin-coated silver nanoparticles. Appl. Biochem. Biotechnol..

[58] Menon S, Rajeshkumar S, Venkat Kumar S (2017). A review on biogenic synthesis of gold nanoparticles, characterization, and its applications. Resour. Eff. Technol..

[59] Saravanan M (2020). Emerging antineoplastic biogenic gold nanomaterials for breast cancer therapeutics: A systematic review. Int. J. Nanomed..

[60] Huang H, Yang X (2004). Synthesis of polysaccharide-stabilized gold and silver nanoparticles: a green method. Carbohydr. Res..

[61] Smitha SL, Philip D, Gopchandran KG (2009). Green synthesis of gold nanoparticles using *Cinnamomum zeylanicum* leaf broth. Spectrochim. Acta Part A Mol. Biomol. Spectrosc..

[62] Chhatre A, Solasa P, Sakle S, Thaokar R, Mehra A (2012). Color and surface plasmon effects in nanoparticle systems: Case of silver nanoparticles prepared by microemulsion route. Colloids Surf. A Physicochem. Eng. Asp..

[63] Turkevich J, Stevenson PC, Hillier J (1951). A study of the nucleation and growth processes in the synthesis of colloidal gold. Discuss. Faraday Soc..

[64] Faraday M (1857). X. The Bakerian Lecture.—Experimental relations of gold (and other metals) to light. Philos. Trans. R. Soc..

[65] Amendola V, Pilot R, Frasconi M, Maragò OM, Iatì MA (2017). Surface plasmon resonance in gold nanoparticles: A review. J. Phys. Condens. Matter.

[66] Tarasenko N, Butsen A, Nevar E, Savastenko N (2006). Synthesis of nanosized particles during laser ablation of gold in water. Appl. Surf. Sci..

[67] Szunerits S, Boukherroub R (2006). Electrochemical investigation of gold/silica thin film interfaces for electrochemical surface plasmon resonance studies. Electrochem. Commun..

[68] Gharibshahi E, Saion E (2012). Influence of dose on particle size and optical properties of colloidal platinum nanoparticles. Int. J. Mol. Sci..

[69] Yadi M (2018). Current developments in green synthesis of metallic nanoparticles using plant extracts: A review. Artif. Cells Nanomed. Biotechnol..

[70] Alkilany AM, Murphy CJ (2010). Toxicity and cellular uptake of gold nanoparticles: What we have learned so far?. J. Nanopart. Res..

[71] Conner SD, Schmid SL (2003). Regulated portals of entry into the cell. Nature.

[72] Kang MS, Lee SY, Kim KS, Han D-W (2020). State of the art biocompatible gold nanoparticles for cancer theragnosis. Pharmaceutics.

[73] Benedec D (2018). *Origanum vulgare* mediated green synthesis of biocompatible gold nanoparticles simultaneously possessing plasmonic, antioxidant and antimicrobial properties. Int. J. Nanomed..

[74] Vimbela GV, Ngo SM, Fraze C, Yang L, Stout DA (2017). Antibacterial properties and toxicity from metallic nanomaterials. Int. J. Nanomed..

[75] Sathiyaraj S (2021). Biosynthesis, characterization, and antibacterial activity of gold nanoparticles. J. Infect. Public Health.

[76] Bruna T, Maldonado-Bravo F, Jara P, Caro N (2021). Silver nanoparticles and their antibacterial applications. Int. J. Mol. Sci..

[77] Arumai Selvan D, Mahendiran D, Senthil Kumar R, Kalilur Rahiman A (2018). Garlic, green tea and turmeric extracts-mediated green synthesis of silver nanoparticles: Phytochemical, antioxidant and in vitro cytotoxicity studies. J. Photochem. Photobiol. B.

[78] Looi CY (2013). Induction of apoptosis in human breast cancer cells via caspase pathway by vernodalin isolated from *Centratherum anthelminticum* (L.) seeds. PLoS ONE.

[79] Yang Y (2019). Mechanism of cell death induced by silica nanoparticles in hepatocyte cells is by apoptosis. Int. J. Mol. Med..

[80] Al-kawmani AA (2020). Apoptosis-inducing potential of biosynthesized silver nanoparticles in breast cancer cells. J. King Saud Univ. Sci..

[81] Landgraf L (2015). Comparative evaluation of the impact on endothelial cells induced by different nanoparticle structures and functionalization. Beilstein J. Nanotechnol..

[82] Jiang W, Kim BYS, Rutka JT, Chan WCW (2008). Nanoparticle-mediated cellular response is size-dependent. Nat. Nanotechnol..

[83] Liu Z (2014). Effects of internalized gold nanoparticles with respect to cytotoxicity and invasion activity in lung cancer cells. PLoS ONE.

[84] Favi PM (2015). Shape and surface effects on the cytotoxicity of nanoparticles: Gold nanospheres versus gold nanostars. J. Biomed. Mater. Res. A.

[85] Afrooz ARMN (2013). Spheres versus rods: The shape of gold nanoparticles influences aggregation and deposition behavior. Chemosphere.

[86] Tang Y (2015). In vitro cytotoxicity of gold nanorods in A549 cells. Environ. Toxicol. Pharmacol..

[87] Amarnath K, Mathew NL, Nellore J, Siddarth CR, Kumar J (2011). Facile synthesis of biocompatible gold nanoparticles from *Vites vinefera* and its cellular internalization against HBL-100 cells. Cancer Nanotechnol..

[88] Barabadi H (2019). A systematic review of the genotoxicity and antigenotoxicity of biologically synthesized metallic nanomaterials: Are green nanoparticles safe enough for clinical marketing?. Medicina (Kaunas).

[89] Choudhury D (2013). Unprecedented inhibition of tubulin polymerization directed by gold nanoparticles inducing cell cycle arrest and apoptosis. Nanoscale.

[90] Selim ME, Hendi AA (2012). Gold nanoparticles induce apoptosis in MCF-7 human breast cancer cells. Asian Pac. J. Cancer Prev..

