# Supplementary material for: Green synthesis of novel stable biogenic gold nanoparticles for breast cancer therapeutics via the induction of extrinsic and intrinsic pathways
Source: Sci Rep. 2022 Jul 7;12:11518. doi: 10.1038/s41598-022-15648-y (PMC9262950; doi:10.1038/s41598-022-15648-y)
Supplement: Supplementary file 6 — Supplementary Information 6. [file 41598_2022_15648_MOESM6_ESM.pdf]

MCF-7 cells treated with the designated concentration of the tested AuNPs were incubated to test the effect of the treatment on the gene expression levels of three genes Bcl2, IKap-α, and Survivin genes. In addition, GAPDH was used as a housekeeping gene (GAPDH gene) control.
The experiment was performed 3 times for each treatment and each one was treated independently from the other one.
Treated and untreated control cells were subjected to RNA extraction using Qiagen RNeasy extraction Kit (Qiagen, Germany) DNA was removed by DNase I according to the manufacturer.1.0 µg of the DNA-free RNA was used for cDNA synthesis using Oligo-dT primer and AMV reverse transcriptase (Promega Corp., Madison, WI). Serial dilution was performed from the cDNA and 1.0 µl was used as a template in RT-PCR optimization. 1:5 (cDNA: Nuclease free water) cDNA dilution was the best ratio for RTq-PCR reactions. Gene specific primer sets were used to quantify the expression level of each gene. The RTq-PCR reaction contained 1µl of cDNA (1:5), 5.0 p.mol of each primer, 10.0 ul 2X SYBR green reaction mix (QuantiTect SYBR Green PCR Kits), and nuclease free water up to 20 ul. The reaction was done using Light Cycler fluorimeter BIORAD S1000 Tm thermal cycler (BioRad, USA). The PCR cycling program was as follows

Initial cycle: 95C°/12 min
40 cycles: 95C°/15 sec, 60 C°/20 sec, 72 C°/20 sec
Plate read

Data analysis was performed using CFX 3.1 software.
The average C_t value of each treatment (three replicates) was recorded as the mean of C_t values.

Gene expression was normalized to housekeeping gene as follows;
Normalized $C_t = 2^{-\Delta C_t}$, where $\Delta C_t = (C_{t\_Target} - C_{t\_HKgene})$

Fold change calculation was obtained from the ratio between the normalized C_t treatment and the C_t of the corresponding control.
